# Altered cfDNA fragmentation profile in hypomethylated regions as diagnostic markers in breast cancer

**DOI:** 10.1186/s13072-023-00508-4

**Published:** 2023-09-23

**Authors:** Jun Wang, Yanqin Niu, Ming Yang, Lirong Shu, Hongxian Wang, Xiaoqian Wu, Yaqin He, Peng Chen, Guocheng Zhong, Zhixiong Tang, Shasha Zhang, Qianwen Guo, Yun Wang, Li Yu, Deming Gou

**Affiliations:** 1 Shenzhen Key Laboratory of Microbial Genetic Engineering, Vascular Disease Research Center, College of Life Sciences and Oceanography, Guangdong Provincial Key Laboratory of Regional Immunity and Disease, Carson International Cancer Center, School of Medicine, Shenzhen University, Shenzhen, 518060 China; 2 Department of Thyroid and Breast, Huazhong University of Science and Technology Union Shenzhen Hospital, Shenzhen, 518052 China; 3 Surgical Department, General Hospital of Ningxia Medical University, Yinchuan, 750003 China; 4 Department of Hematology and Oncology, Shenzhen University General Hospital, Carson International Cancer Research Center, School of Medicine, Shenzhen University, Shenzhen, 518060 China

## Abstract

**Background:**

Breast cancer, the most common malignancy in women worldwide, has been proven to have both altered plasma cell-free DNA (cfDNA) methylation and fragmentation profiles. Nevertheless, simultaneously detecting both of them for breast cancer diagnosis has never been reported. Moreover, although fragmentation pattern of cfDNA is determined by nuclease digestion of chromatin, structure of which may be affected by DNA methylation, whether cfDNA methylation and fragmentation are biologically related or not still remains unclear.

**Methods:**

Improved cfMeDIP-seq were utilized to characterize both cfDNA methylation and fragmentation profiles in 49 plasma samples from both healthy individuals and patients with breast cancer. The feasibility of using cfDNA fragmentation profile in hypo- and hypermethylated regions as diagnostic markers for breast cancer was evaluated.

**Results:**

Mean size of cfDNA fragments (100–220 bp) mapped to hypomethylated regions decreased more in patients with breast cancer (4.60 bp, 172.33 to 167.73 bp) than in healthy individuals (2.87 bp, 174.54 to 171.67 bp). Furthermore, proportion of short cfDNA fragments (100–150 bp) in hypomethylated regions when compared with it in hypermethylated regions was found to increase more in patients with breast cancer in two independent discovery cohort. The feasibility of using abnormality of short cfDNA fragments ratio in hypomethylated genomic regions for breast cancer diagnosis in validation cohort was evaluated. 7 out of 11 patients were detected as having breast cancer (63.6% sensitivity), whereas no healthy individuals were mis-detected (100% specificity).

**Conclusion:**

We identified enriched short cfDNA fragments after 5mC-immunoprecipitation (IP) in patients with breast cancer, and demonstrated the enriched short cfDNA fragments might originated from hypomethylated genomic regions. Furthermore, we proved the feasibility of using differentially methylated regions (DMRs)-dependent cfDNA fragmentation profile for breast cancer diagnosis.

**Supplementary Information:**

The online version contains supplementary material available at 10.1186/s13072-023-00508-4.

## Introduction

Breast cancer is the most commonly diagnosed malignancy in women worldwide with more than 2.3 million new cases and 690,000 deaths each year [1]. Early detection of breast cancer is crucial for improving prognosis and survival [2]. Therefore, the development of minimally invasive biomarkers to facilitate early diagnosis has become a major focus of research. Cell-free DNA (cfDNA) in blood has emerged as a promising biomarker for early diagnosis and monitoring progression of cancer [3–6]. The current research on cfDNA-based cancer detection approaches mainly focus on identifying the differences of methylation or fragment size between cancer- and noncancer-derived cfDNA, which appear at an early phase of carcinogenesis [7–11].

Global hypomethylation along with hypermethylation of tumor suppressor genes have been demonstrated to be present in breast cancer [12, 13]. Altered methylation of specific genes in cfDNA could serve as biomarkers for early diagnosis have also been widely reported [14, 15]. In addition, cancer-derived cfDNA fragments were proved to be shorter than noncancer-derived cfDNA fragments, which led to the aberrant size distribution of cfDNA fragments in patients with cancer [7, 10, 11, 16]. Genome-wide cfDNA fragmentation profiling was further reported to achieve 70% detecting sensitivity with 95% specificity as biomarker for breast cancer diagnosis [7].

These studies suggested that abnormal methylation and fragmentation were present in cancer-derived cfDNA. Conceptually, approaches that simultaneously detecting these abnormalities can better differentiate the origin of cfDNA, and improve cancer detection efficacy.

Because cfDNA is originated from the nucleases digestion of chromatin during multiple cellular processes including apoptosis, necrosis and active cellular secretion [17], fragmentation pattern of cfDNA should be closely related to the accessibility of chromatin. Epigenetic modification, nucleosome position and location of transcription machinery have been characterized to affect the structure of chromatin [16–20]. Therefore, we hypothesized that methylation profile of cfDNA, which had implications for chromatin remodeling, should be related to fragmentation profile of cfDNA.

Recently the studies have revealed important connections between DNA methylation patterns and cfDNA fragmentation characteristics. It was demonstrated that DNA methylation regulates nuclease cutting preferences during apoptosis, affecting cfDNA fragment size distribution [21]. Furthermore, studies showed that cleavage patterns surrounding CpG dinucleotides reflect regional cfDNA methylation levels [22]. Collectively, these findings suggest DNA methylation is a key molecular regulator of cfDNA fragmentation. However, the interplay between methylation patterns and fragmentation in cfDNA from both breast cancer patients and healthy individuals has not been fully elucidated.

**Fig. 1:**
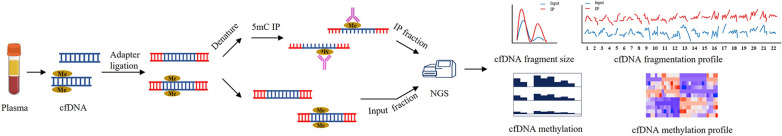
Schematic representation of the improved cfMeDIP-seq approach. Plasma was collected from patients with breast cancer and healthy individuals. cfDNA was extracted and processed with adapter ligation and 5mC-immunoprecipitation (IP) for sequencing library construction. cfDNA methylation and fragmentation profile were identified through analyzing the NGS data

In this study, we used the improved cfMeDIP-seq approach to investigate whether the aberrant methylation of cfDNA in patients with breast cancer was related to cfDNA fragmentation profile or not (Fig. 1).In addition, we further evaluated the possibility of detecting both methylation and fragmentation profile of cfDNA for better detecting efficacy of breast cancer.

## Results

### Altered cfDNA fragmentation profile upon 5mC-immunoprecipitation (IP)

**Fig. 2:**
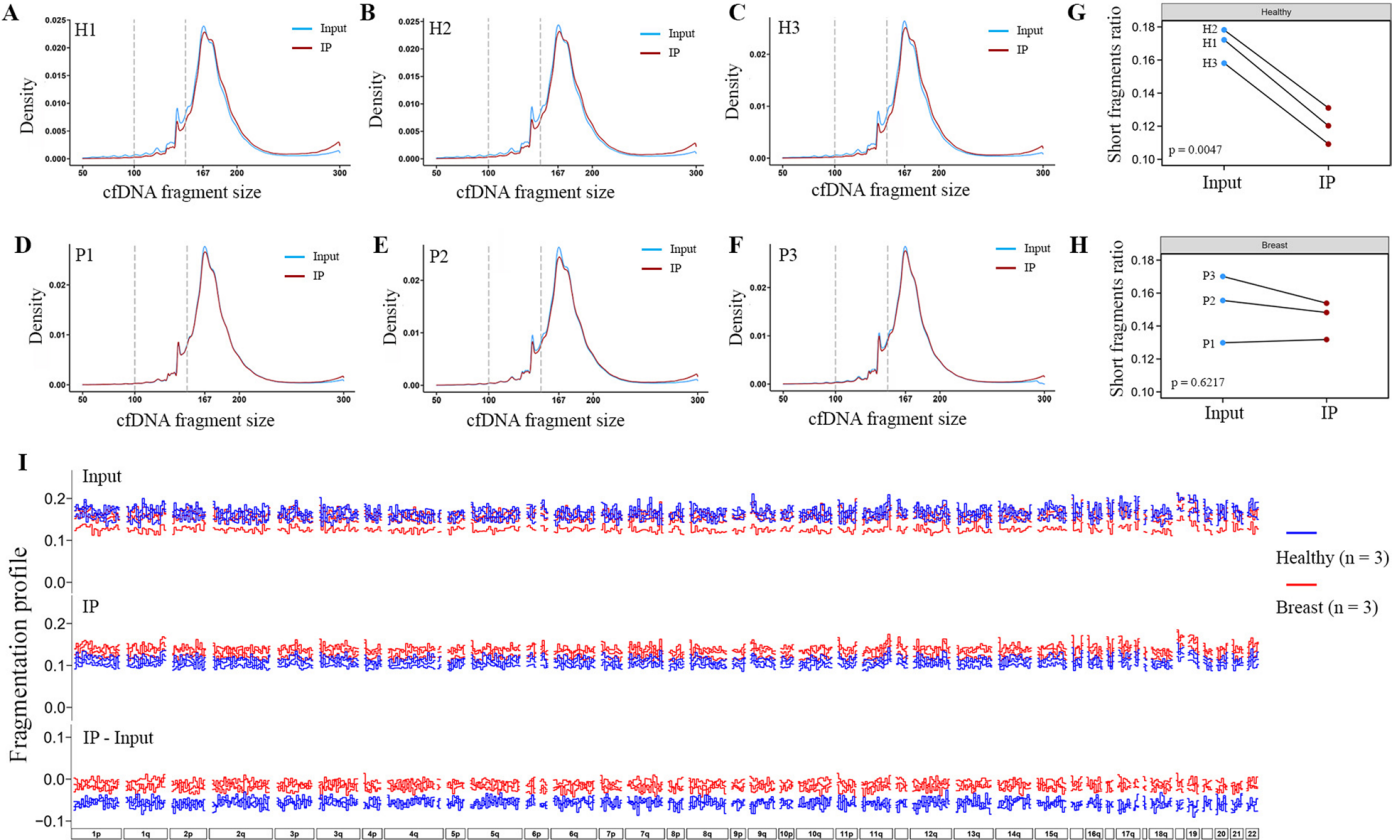
Altered fragmentation profiles of methylated cfDNA in patients with breast cancer. **A–F** Distribution of cfDNA fragment size in Input library (blue line) and IP library (red line) were shown for healthy individuals (H1, H2, and H3) (**A**–**C**), and patients with breast cancer (P1, P2, and P3) (**D**–**F**). The vertical dashed line indicated cfDNA fragment size at 100 bp and 150 bp. **G**, **H** Evaluation of short cfDNA fragments ratio changes (defined as the ratio of short cfDNA fragments (100–150 bp) to long cfDNA fragments (151–220 bp)) in IP libraries when compared with corresponding Input libraries were shown for healthy individuals (*p* = 0.0047, 0.1202 ± 0.0109 vs. 0.1695 ± 0.0103) (**G**) and patients with breast cancer (*p* = 0.6217, 0.1446 ± 0.0115 vs. 0.1518 ± 0.0204) (**H**) in discovery cohort 1. **I** Genome-wide cfDNA fragmentation profiles (the ratio of short cfDNA fragments (100–150 bp) to long cfDNA fragments (151–220 bp)) in Input (upper panel) and IP (middle panel) libraries were shown in 5-Mb windows for patients with breast cancer (red, *N* = 3) and healthy individuals (blue, *N* = 3), changes of cfDNA fragmentation profile (IP-Input, lower panel) were calculated through subtracting the short fragments ratio in Input libraries format in IP libraries and shown in each 5-Mb window. Healthy, healthy individuals; Breast, patients with breast cancer

We utilized improved cfMeDIP-seq method with newly designed multiplexed adapter containing molecular barcode to remove the PCR duplicate (Additional file 1: Table S6) in this study. As cancer-derived cfDNA fragments have been reported to exhibit altered methylation and smaller size when compared with noncancer-derived cfDNA fragments [3, 10], we focused our analysis on cfDNA fragments ranging from 100 to 220 base pairs (bp), which allowed us to investigate whether the release of cancer-derived cfDNA was related to DNA methylation or not. In a preliminary analysis of discovery cohort 1, cfDNA extracted from plasma of 3 healthy individuals (H1, H2, and H3) and 3 breast carcinoma patients (P1, P2, and P3) in recovery period post-surgery with relatively low tumor burden were used for cfMeDIP-seq library construction with modifications (Table 1, Additional file 2: Fig. S1A–E, and Additional file 1: Table S1). Input and IP libraries were sequenced for pair-end reads with around 0.5 $\times$ and 5 $\times$ coverage respectively (Additional file 1: Table S2). Interestingly, we observed a significant decrease of short cfDNA fragments (100–150 bp) density and ratio (defined as the ratio of short cfDNA fragments to long cfDNA fragments (151–220 bp)) in IP libraries when compared with it in corresponding Input libraries for healthy individuals (Fig. 2A–C and G), whereas these phenomena were not seen in patients with breast cancer (Fig. 2D–F and H). Furthermore, mean cfDNA fragments size was found to increase from 170.06 (Input libraries) to 173.04 (IP libraries) bp in healthy individuals, which was not observed in patients with breast cancer (170.51 to 170.71 bp) as well (Additional file 2: Fig. S2A, B). To examine differences between healthy individuals and patients with breast cancer, change of short fragments ratio from IP library to corresponding Input library was calculated, we found smaller changes in patients with breast cancer compared with healthy individuals (Additional file 2: Fig. S2C–E and Additional file 1: Table S3).

**Table 1:** Clinical information of the participants in this study

	Discover cohort 1	Discovery cohort 2	Validation cohort
*n*	Breast^a^(*N* = 3) Healthy^b^(*N* = 3)	Breast (*N* = 12) Healthy (*N* = 12)	Breast (*N* = 11) Healthy (*N* = 8)
Age, years	Breast (49.3 42–54) Healthy (47.7, 42–51)	Breast (58.2 45–70) Healthy (54.5, 41–72)	Breast (51.5, 26–71) Healthy (48.0, 25–57)
Gender:female	Breast (3, 100%) Healthy (3, 100%)	Breast (12, 100%) Healthy (12, 100%)	Breast (12, 100%) Healthy (8, 100%)
Stages	Breast (Post-sugery D393, 1, 33%; Post-surgery D726, 1, 33%; Post-surgery D289, 1, 33%) Healthy (N.A.)	Breast (I, 3, 25%; II, 5, 42%; III, 2, 17%; IV, 2, 17%) Healthy (N.A.)	Breast (I, 7, 64%; II, 3, 27%; III, 1, 9%) Healthy (N.A.)

^a^Breast in this table indicates patients with breast cancer

^b^Healthy in this table indicates healthy individuals

To find out variation of the short fragments ratio across human genome, cfDNA fragmentation profiles in both Input (Fig. 2I, upper panel) and IP (Fig. 2I, middle panel) libraries were shown in 5-Mb windows for participants in discovery cohort 1 according to the method described previously [7]. Changes of cfDNA fragmentation profile (IP-Input) due to 5mC-IP were calculated through subtracting the short fragments ratio in Input libraries from it in IP libraries across each 5-Mb genomic window (Fig. 2I, lower panel). Smaller changes of short fragments ratio between IP library and Input library were observed in almost all genomic windows across human genome for patients with breast cancer. Overall, these results suggested that more short cfDNA fragments in breast cancer patients were enriched during 5mC-IP.

### Relationship between methylation and fragment size in cfDNA

**Fig. 3:**
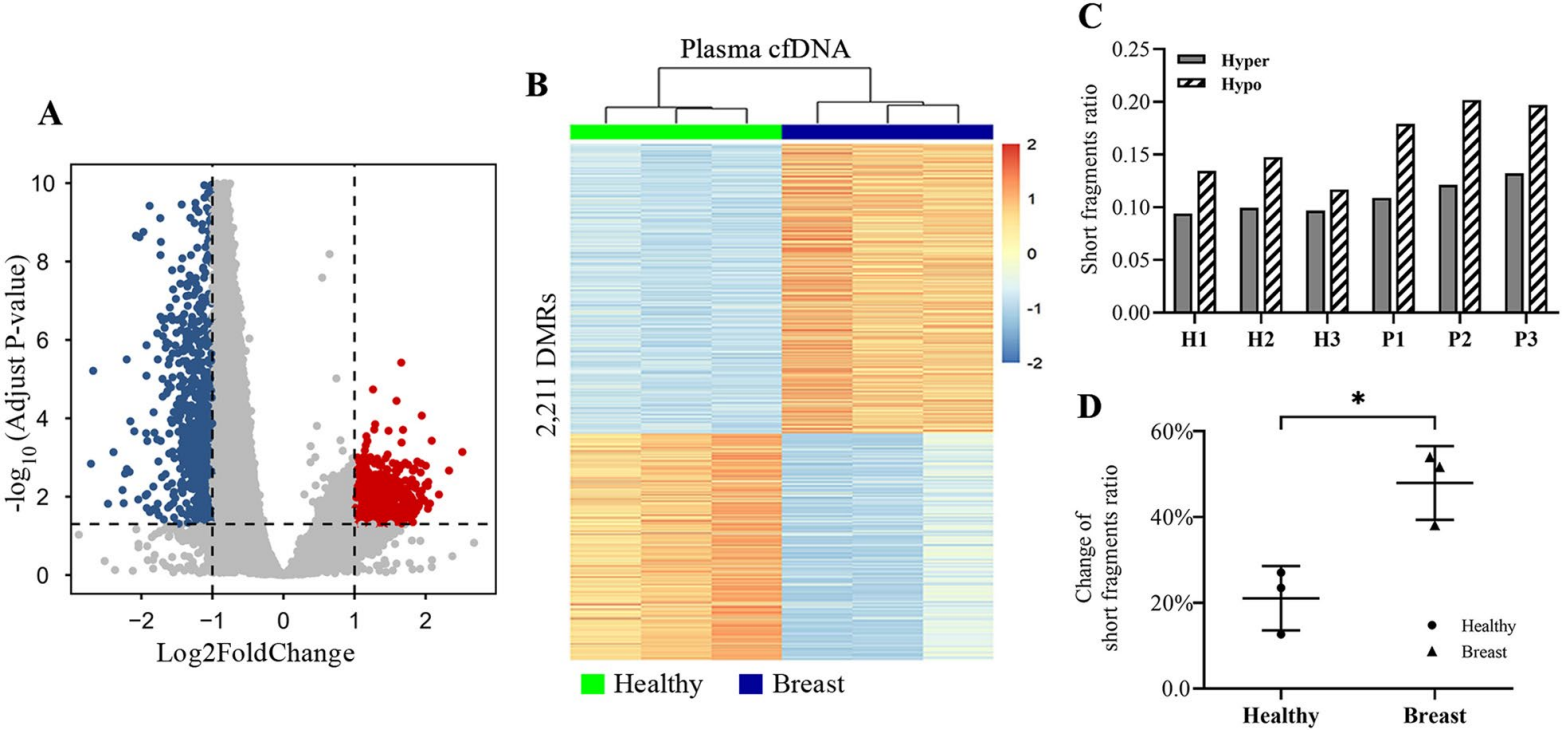
Short cfDNA fragments ratio among DMRs in discovery cohort 1. **A** Volcano plot of DMRs from patients with breast cancer (*N* = 3) versus healthy individuals (*N* = 3). Significantly hypermethylated windows were highlighted in red dots with padj < 0.05, log2Foldchange > 1, significantly hypomethylated windows were highlighted in blue dots with padj < 0.05 and log2FoldChange < − 1. **B** Heatmap of the 2,211 DMRs identified in plasma cfDNA from patients with breast cancer and healthy individuals. **C** Short fragments ratio of cfDNA in hypermethylated (H1: 0.0940; H2: 0.00994; H3: 0.0967; P1: 0.1089; P2: 0.1213; P3: 0.1322) and hypomethylated (H1: 0.1344; H2:0.1477; H3: 0.1167; P1: 0.1790; P2: 0.2017; P3: 0.1970) regions in patients with breast cancer and healthy individuals. **D** Percentage change of short fragments ratio in hypomethylated regions compared with it in hypermethylated regions for patients with breast cancer and healthy individuals. Hyper, hypermethylated genomic regions; Hypo, hypomethylated genomic regions; Healthy, healthy individuals; Breast, patients with breast cancer; * represents *p* value < 0.05

To examine the relationship between enriched short cfDNA fragments and DNA methylation in patients with breast cancer, we first identified 2211 differentially methylated regions (DMRs) in cfDNA between patients with breast cancer and healthy individuals (1241 hypermethylated, 970 hypomethylated in patients at padj < 0.05 and |log2FoldChange|> 1 with each region represented 10 kb genomic window) (Fig. 3A, B, and Additional file 1: Table S4). We then evaluated DMRs-dependent cfDNA fragmentation pattern in IP libraries, it was found that cfDNA released from hypomethylated regions had higher short fragments ratio than hypermethylated regions in both patients and healthy individuals (Fig. 3C). Further analysis showed patients with breast cancer had greater percentage change of short fragments ratio in hypomethylated regions compared with it in hypermethylated regions (Fig. 3D), which indicated that the enriched short cfDNA fragments might be mainly released from hypomethylated regions.

**Fig. 4:**
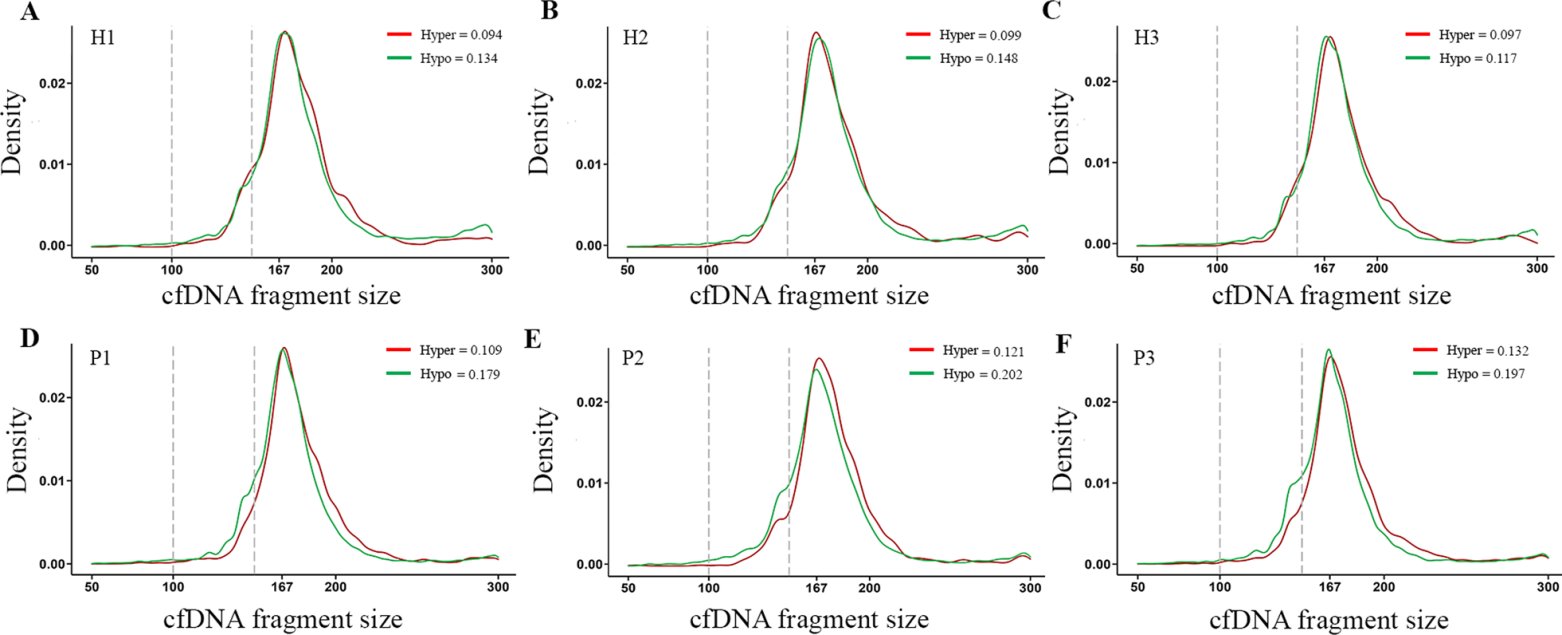
Altered cfDNA fragmentation patterns in hypomethylated regions in patients with breast cancer. **A–C** Distribution of cfDNA fragment size was shown for healthy individuals H1, H2, and H3 in hypermethylated genomic regions (red) and hypomethylated genomic regions (green). **D–F** Distribution of cfDNA fragment size was shown for patients with breast cancer P1, P2, and P3 in hypermethylated genomic regions (red) and hypomethylated genomic regions (green). The vertical dashed line indicated cfDNA fragment size at 100 bp and 150 bp

In accordance with increased short fragments ratio in hypomethylated regions, size distribution of cfDNA fragments mapped to hypomethylated regions was found to shift to the direction of smaller size compared with cfDNA fragments mapped to hypermethylated regions, and this shift was to a greater extent in patients with breast cancer (Fig. 4A–F). Moreover, mean size of cfDNA fragments mapped to hypomethylated regions decreased more in patients with breast cancer (4.60 bp, 172.33 bp in hypermethylated regions to 167.73 bp in hypomethylated regions) than healthy individuals (2.87 bp, 174.54 bp in hypermethylated regions to 171.67 bp in hypomethylated regions).

To further confirm the changes of short fragments ratio in patients with breast cancer, we evaluated the cfDNA fragmentation profile in hyper- and hypomethylated regions in another discovery cohort 2 (*N* = 24, Table 1). Patients with breast cancer (*N* = 12) in this cohort had not undergone previous treatment and were confirmed through biopsy. We identified 5148 DMRs with 3002 hypermethylated and 2146 hypomethylated (Additional file 2: Fig. S3A). 9 out of 12 patients with breast cancer showed increased short fragments ratio in hypomethylated regions compared with it in hypermethylated regions. Only 3 out of 12 healthy individuals showed the similar increase, whereas most of the other healthy individuals remain unchanged (Additional file 2: Fig. S3B). Moreover, patients with breast cancer also had greater percentage change of short fragments ratio in hypomethylated regions compared with it in hypermethylated regions (Additional file 2: Fig. S3C), which was consistent with the results in discover cohort 1.

Collectively, these findings again demonstrated that in contrast to healthy individuals, patients with breast cancer had enriched short cfDNA fragments during 5mC-IP reaction, which might mainly originated from hypomethylated genomic regions. In addition, to further validate the origin of short cfDNA fragments, size distribution of cfDNA fragments in patients with lung cancer from another study (E-MTAB-7163) were also investigated [23]. As expected, patients with lung cancer had higher percentage change of short fragments ratio in hypomethylated regions compared with it in hypermethylated regions (Additional file 2: Fig. S4A, B).

### DMRs-dependent cfDNA fragmentation profiles of breast cancer

**Fig. 5:**
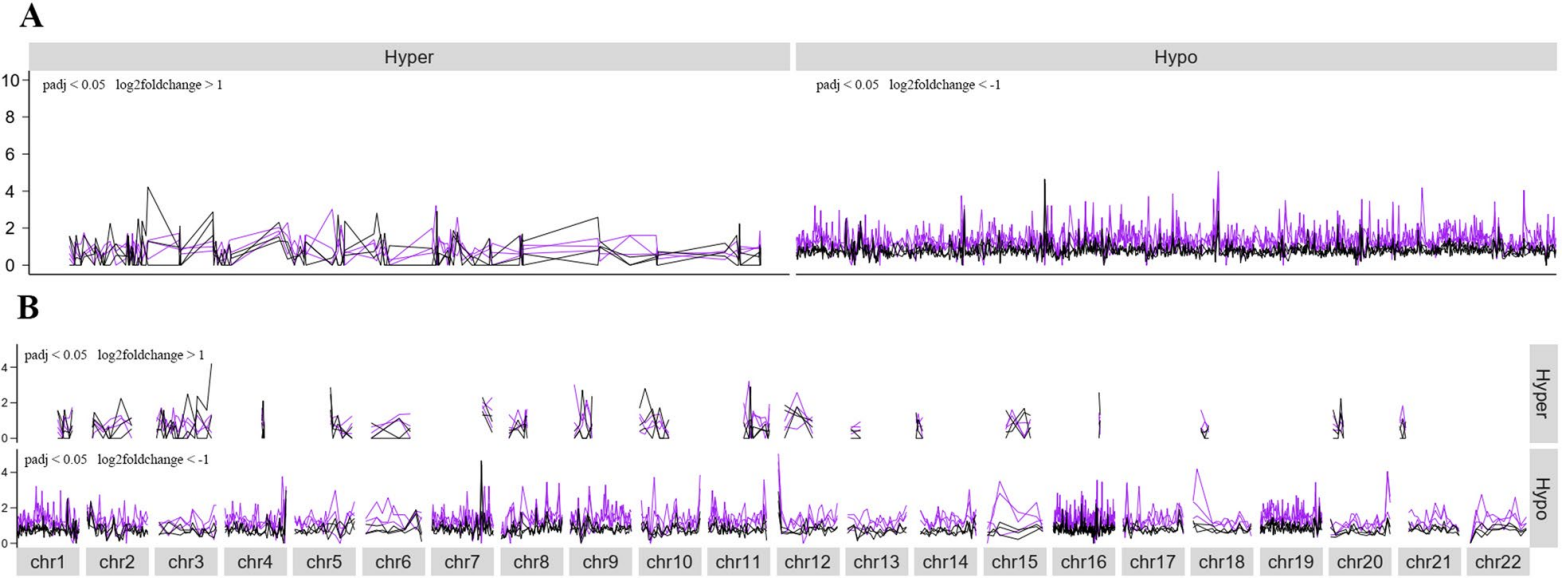
DMRs-dependent cfDNA fragmentation profiles. **A** Input-adjusted short fragments ratio were shown with 10 kb windows in hypermethylated and hypomethylated regions for both patients with breast cancer (purple) and healthy individuals (black). **B** Distribution of the cfDNA fragmentation profile mentioned above was shown across human genome. The input-adjusted short fragments ratio in each 10 kb window was calculated by dividing short fragments ratio in each 10 kb window by it in corresponding input libraries. Differentially methylated 10 kb windows were selected for representation according to the following criteria: (1) hypermethylated 10 kb windows have padj < 0.05 and log2FoldChange > 1; (2) hypomethylated 10 kb windows have padj < 0.05 and log2FoldChange < − 1; (3) the selected windows should have at least 20 deduplicated cfDNA fragments for all samples including patients with breast cancer and healthy individuals; (4) the selected windows should have input-adjusted short fragments ratio of less than 10 for any samples analyzed. Hyper, hypermethylated genomic regions; Hypo, hypomethylated genomic regions

To investigate the utility of integrating methylation and fragmentation data for breast cancer diagnosis, we evaluated the feasibility of applying DMRs-dependent cfDNA fragmentation profiles to distinguish cancer patients from healthy individuals in the discovery cohort 1. We analyzed cfDNA fragmentation across multiple genomic window sizes (300 bp, 500 bp, 1 kb, 2 kb, 5 kb and 10 kb) to identify the optimal range for concurrently characterizing methylation patterns and fragmentation. We found 10 kb windows provided sufficient resolution to delineate DMRs while retaining adequate cfDNA fragments to reliably quantify short fragment ratios. To account for potential biases contributed by short fragments prior to 5mC-IP, the short fragments ratio in IP libraries was normalized by it in corresponding input libraries across each 10 kb DMR window. This input-adjusted short fragments ratio was calculated for 93 hypermethylated genomic windows and 691 hypomethylated genomic windows, defined as having at least 20 nonduplicated cfDNA fragments across all samples and the input-adjusted short fragments ratio of below 10. As expected, the input-adjusted short fragments ratio in hypomethylated genomic windows could differentiate cancer patients from healthy individuals, which was rarely observed in hypermethylated genomic windows (Fig. 5A, and Additional file 2: Fig. S5A). Similar discriminatory patterns were continuously evident even with progressively decreasing DMR calling thresholds (padj < 0.05 and |log2FoldChange|> 0.9 to padj < 0.05 and |log2FoldChange|> 0.5) (Additional file 2: Fig. S6). Moreover, hypomethylated windows with diagnostic fragmentation profiles were distributed across nearly all chromosomes (Fig. 5B, and Additional file 2: Fig. S5B). These findings suggested that variation in DMRs-dependent cfDNA fragmentation profile could differentiate patients with breast cancer from healthy individuals.

### Breast cancer diagnostic accuracy in validation cohort

To verify whether the findings obtained from discovery cohort could be applied for diagnosis of breast cancer, we performed cfMeDIP-seq for cfDNA extracted from 11 patients with breast cancer (P4–P14) and 8 healthy individuals (H4–H11) in validation cohort (Table 1, Additional file 1: Table S1). All patients in this cohort with breast cancer had not undergone previous treatment and were confirmed through biopsy. Similarly, increased short cfDNA fragments density in IP libraries of patients with breast cancer was observed (Additional file 2: Fig. S7A, B and Additional file 2: Fig. S8). Within the identified 731 DMRs, greater percentage change of short fragments ratio as well as shift of size distribution of cfDNA fragments in hypomethylated regions when compared with hypermethylated regions were also found for patients with breast cancer (Additional file 2: Fig. S9A–D, 10, and Additional file 1: Table S5).

Subsequently, we assessed whether DMRs-dependent cfDNA fragmentation profile could differentiate cancer patients from healthy individuals in validation cohort. It was found that abnormal input-adjusted short fragments ratio in specific hypomethylated genomic windows were present for most of the patients with breast cancer, whereas it remained consistent in healthy individuals (Additional file 2: Fig. S11, 12).

**Fig. 6:**
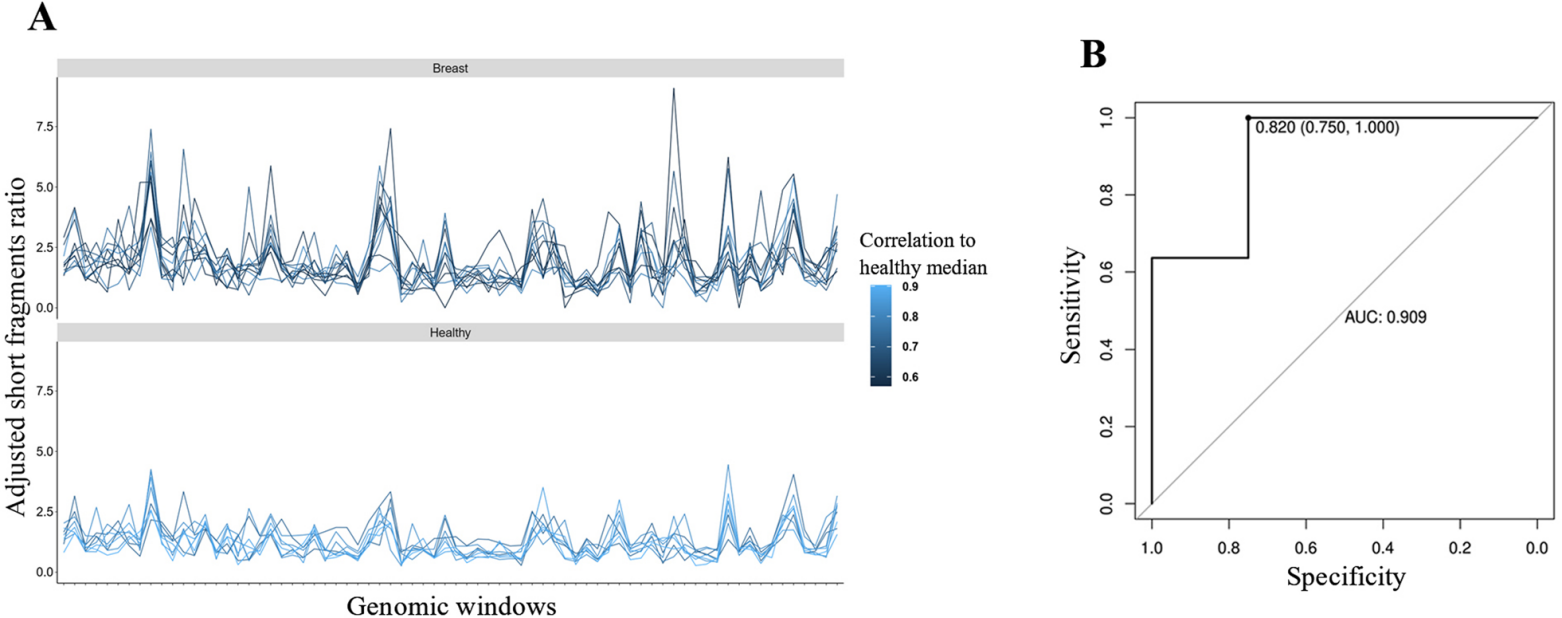
Detection of breast cancer using DMRs-dependent cfDNA fragmentation profile. **A** Input-adjusted short fragments ratio was depicted for hypomethylated genomic windows, individual profile was colored according to their Pearson correlation to the healthy median in each genomic window. **B** Receiver operator characteristics for breast cancer detection using correlation assessment of DMRs-dependent cfDNA fragmentation profile. AUC = 0.909; 95% CI (0.771–1.000). Healthy, healthy individuals; Breast, patients with breast cancer

We then developed an approach called ‘correlation assessment of DMRs-dependent cfDNA fragmentation profile’ to evaluate the abnormality of short fragments ratio in 72 frequently altered hypomethylated genomic windows with at least 20 unduplicated cfDNA fragments for all samples and input-adjusted short fragments ratio of no more than 10 for any samples within each window. Correlation analysis of input-adjusted short fragments ratio in the 72 hypomethylated windows of each participant to the median of it from healthy individuals was performed. It was found that healthy individuals had higher correlation with an average of 0.83, whereas patients with breast cancer had lower correlation with an average of 0.68 (Fig. 6A). If using the correlation value as classifier for detecting patients as being healthy or having cancer, we could detected 7 out of 11 patients as having breast cancer (63.6% sensitivity) at a threshold of 0.72,, whereas no healthy individuals were mis-detected (100% specificity) (Table 2). Receiver operator characteristic analysis for the detection of patients with cancer had an area under the curve (AUC) value of 0.909 (95% confidence interval, 0.771–1.000) (Fig. 6B). Taken together, DMRs-dependent cfDNA fragmentation profiling could distinguish patients with breast cancer and healthy individuals.

**Table 2:** Effect of cut-offs threshold in detecting breast cancer in validation cohort

Cut-offs (correlation to Healthy median)	Sensitivity (%)	Specificity (%)
0.82	100.0%	75.0%
0.80	90.9%	75.0%
0.76	72.7%	75.0%
0.72	63.6%	100.0%

## Discussion

Genome-wide DNA methylation alterations have been demonstrated to occur in neoplastic tissue, leading to changes of chromatin structure [24, 25], which is the direct source in releasing cfDNA into plasma. Although it is known DNA methylation impacts cfDNA release, the extent of this effect remains unclear. Our findings that short cfDNA fragments preferentially originate from hypomethylated regions in breast cancer patients is consistent with recent studies elucidating connections between DNA methylation and cfDNA fragmentation [21]. Furthermore, our study suggested that DMRs-dependent cfDNA fragmentation profile may provide an alternative approach for breast cancer diagnosis.

**Fig. 7:**
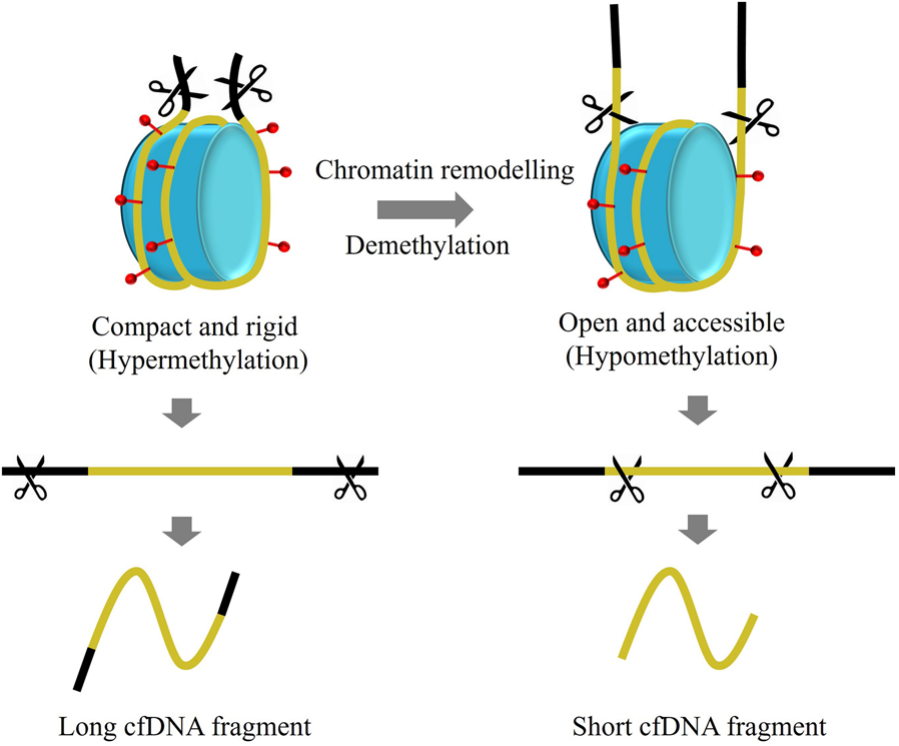
Illustration of the potential relationship between cfDNA methylation and fragment size. Nucleosomes with wrapped DNA (yellow line) exist as compact and rigid structure under normal physiological conditions, cfDNA is released through nuclease digestion (scissors) of the linker sequences (black line) and thus produce long cfDNA fragments. During cancer development, chromatin remodeling and DNA demethylation lead to global hypomethylation, and the decreased DNA methylation level result open and accessible wrapped DNA in nucleosomes, therefore, nuclease digestion during apoptosis produce short cfDNA fragments

Although the recent studies have revealed that cancer-derived cfDNA fragments tend to be shorter as compared to noncancer-derived cfDNA [10, 11], the underlying molecular mechanisms governing this size reduction are still under investigation and remain to be fully elucidated.. Differences in nucleosome wrapping and nuclease activity during apoptosis were proposed to impact cfDNA fragment size in plasma [26]. As nucleosome compaction and rigidity decrease upon DNA demethylation [27, 28], hypomethylated genomic regions should theoretically be more susceptible to nuclease digestion during apoptosis. In accordance with this hypothesis, An et al. revealed DNA hypomethylation increases nucleosome accessibility, enabling more cutting within nucleosomes to generate shortened cfDNA molecules. The enrichment of short fragments from hypomethylated regions were observed align with this proposed mechanism. Our results also showed that cfDNA fragments originated from hypomethylated regions in patients with breast cancer tend to have significant smaller size compared with healthy individuals, which might be the result of excessive digestion of the wrapped DNA in nucleosome(Fig. 7). Furthermore, decreased methylation level that presented in white blood cells of patients with breast cancer may exacerbate nuclease digestion by reducing chromatin stability and integrity (Fig. 7) [29, 30]. Despite the obvious variation of cfDNA fragmentation profile in hypomethylated regions in patients with breast cancer, it was relatively consistent in healthy individuals. We identified that short fragments ratio of cfDNA mapped to both hypermethylated regions and hypomethylated regions had less changes in healthy individuals, and we supposed this phenomenon was an indicator of genome instability of breast cancer patients compared with healthy individuals.

Hypomethylation of oncogene promoter regions frequently occurs in breast carcinomas [31, 32], suggesting aberrant short cfDNA fragments may partially originate from certain oncogenes. The previous studies suggested that short cfDNA fragments harbor footprints of transcription factors [16]. In this study, cfDNA mapped to TRAF3IP3, PTPRN2 and GALNT9 gene loci in hypomethylated regions exhibited substantially increased short fragments ratio in patients with breast cancer. Upregulation of these three genes during tumor growth has been reported previously, potentially indicating promoter hypomethylation and excessive digestion producing short cfDNA fragments [33–36]. In addition, most hypomethylated windows with altered short fragments ratios colocalized with histone modification marker H3K27ac (data not shown), implying combined effects of DNA and histone modifications on cfDNA fragmentation in breast cancer.

This study showed the possibility of detecting breast cancer through characterizing the fragmentation profile of cfDNA in DMRs. As genome-wide fragmentation profiles varied slightly for participants in validation cohort, differentiating patients with breast cancer from healthy individuals became difficult under this circumstance. While various DMRs were identified, further discrimination of cancer-related from individual variation-related DMRs is needed. Nevertheless, through focusing on DMRs-dependent cfDNA fragmentation profile, we could analyze potentially informative aberrant cfDNA releasing regions and evaluate diagnostic utility of each DMR. With larger validation cohorts, this DMR-directed fragmentation analysis could serve as a companion diagnostic approach.

Aberrant epigenetic modifications, including altered DNA methylation, histone modifications and chromatin remodeling, are considered early events in neoplastic progression [37–40]. Hypomethylated intergenic and intronic regions occurs early in the transition from normal to neoplastic cells [24, 41, 42]. Thus, the release of short cfDNA fragments from hypomethylated regions should manifest at early stages, which enable early and real-time monitoring of breast cancer development through DMRs-dependent cfDNA fragmentation profiling.

Chromatin remodeling involves the assembly of nucleosomes and regulation of DNA accessibility, which may differ depending on the tissue investigated. Calculations of short fragment ratio in DMRs may reflect original chromatin structure and inform tissue of origin [43]. For instance, the altered cfDNA fragmentation profile in TRAF3IP3, PTPRN2 and GALNT9 gene loci, along with their upregulated expression could remind us the chromatin changes due to the development of breast carcinomas. Further characterization of DMRs-dependent cfDNA fragmentation and associated chromatin modifications across cancer types would help validate and extend our findings.

### Conclusions

To summarize, through concurrent analysis of cfDNA methylation and fragment size, this study revealed that short cfDNA fragments were possibly originated from hypomethylated genomic regions in patients with breast cancer. Our approach demonstrated the possibility of using a DMRs-dependent cfDNA fragmentation profiling for breast cancer detection. Several limitations should be taken into consideration. The cohort size in this study was relatively small, thus to avoid misinterpretation, cfDNA samples in discovery cohort 1 were from patients in recovery period, while discovery cohort 2 and validation cohort were from newly diagnosed patients. In searching for differentiated methylation profile between patients with breast cancer and healthy individuals, we identified DMRs as having padj < 0.05 and |log2FoldChange|> 1, which might not be optimal and requires further optimization across cohorts. With larger and more diverse cohorts, refined DMR selection for calculating cfDNA fragment ratios is needed.

## Methods

### Sample collection and cfDNA extraction

Blood samples from patients with breast cancer in discovery cohort 1 (*N* = 3) were obtained at the time of post-surgery D393, D726 and D289 in Shenzhen University General Hospital. Blood samples from patients with breast cancer in discovery cohort 2 (*N* = 12) and validation cohort (*N* = 11) were obtained at the time of diagnosis, before tumor resection or therapy from Huazhong University of Science and Technology Union Shenzhen Hospital. Blood samples from healthy individuals in discovery cohort 1 (*N* = 3), discovery cohort 2 (*N* = 12) and validation cohort (*N* = 8) were obtained at the time of routine screening from Shenzhen University General Hospital and The Third People’s Hospital of Shenzhen, respectively. This study was approved by the Institutional Review Board of Shenzhen University General Hospital and Huazhong University of Science and Technology Union Shenzhen Hospital according to established ethical guidelines as outlined in the Declaration of Helsinki. All patients signed an informed consent document approved by the Institutional Review Board before entering any study. Clinical characteristics for all participants in this study were listed in Table 1 and Additional file 1: Table S1.

All blood samples from participants in this study were collected in tubes containing EDTA as anticoagulant, and processed immediately for plasma isolation. In general, whole blood were first centrifuged at 1000*g* for 10 min at 4 °C for plasma and cellular components separation, and followed by centrifugation at 16000*g* for 10 min at 4 °C for further purifying plasma. The purified plasma was then stored at − 80 °C. cfDNA was extracted from plasma using MiniMaxTM High Efficiency Cell-Free DNA Isolation Kit (Apostle, A17622-250) according to manufacturer’s instructions. The concentration and quality of cfDNA were assessed by the Qubit dsDNA HS Assay kit (Thermo Fisher Scientific, Q32854) and Bioanalyzer 2100 (Agilent Technologies).

### cfMeDIP-seq library construction and sequencing

cfDNA extracted from plasma was then used for cfMeDIP-seq library preparation with the method described previously with the following modifications [3, 44].

(1) ~ 10 to 20 ng cfDNA was ligated with a pool of eight unique adapters with 8-bp molecular barcodes instead of the single adapter (NEBNext Multiplex Oligos for Illumina kit, New England BioLabs) (Additional file 1: Table S6), each initial cfDNA fragment was labeled with a unique barcode. The ligation was conducted by using KAPA Hyper Prep kit (KAPA biosystems, KK8504) according to manufacturer’s instructions; (2) The 5-mC monoclonal antibody (Diagenode, C02010021) immunoprecipitated cfDNA and input cfDNA were amplified using Kapa HiFi Hotstart Mastermix (KAPA biosystems, KK8504) and oligos listed in Additional file 1: Table S6; (3) The multiplexed libraries were subjected for BioAnlyzer analysis before sequencing on Illumina Novaseq platform at HaploX (Shenzhen, China) with 2 × 150-bp paired-end (PE) reads; (4) Input and IP libraries were sequenced at 0.5 × and 5 × respectively.

The specificity of the immunoprecipitation reaction and fold-enrichment ratio in IP libraries were evaluated using the MagMeDIP kit (Diagenode, C02010021) according to the manufacturer’s instructions.

### Data processing and analysis

Raw reads of cfMeDIP-seq Input and IP libraries were processed according to the following steps. (1) Each reads were labeled with the molecular barcode identified in the leading 8-bp sequences of R1 and R2 reads with 1 mismatch allowed, and then the molecular barcode sequences were removed from raw reads. (2) Illumina sequencing adapter and low quality sequences were removed with cutadapter (version 2.10) and trimmomatic (version 0.39), respectively. (3) Paired reads with insert size less than 20 bp were eliminated for further analysis. (4) The remaining reads were aligned against the human reference genome (version hg19) using BWA (version 0.7.17-r1188). (5) Only properly paired and uniquely mapped read pairs were kept, and PCR duplicates defined as having the same genomic start, end and molecular barcode were removed as well. The remaining mapped read pairs in SAM files were converted to BAM format using SAMtools (version 1.7) for further analysis.

### cfDNA fragment size analysis

To calculate fragment size of cfDNA, the bam file obtained above was first processed by R package GenomicAlignments (version 1.24.0), and then a Granges object was generated for calculating the fragment size of each cfDNA molecule by R package GenomicRanges (version 1.40.0). Density plot was generated for illustrating the size distribution of cfDNA fragment through R package ggplot2 (version 3.3.2). Short cfDNA fragments were defined as having lengths between 100 and 150 bp and long fragments as having lengths between 151 and 220 bp according to the previous study [7]. Short fragments ratio was calculated as the counts of short cfDNA fragments mapped to the investigated regions or genomic windows dividing by the counts of long cfDNA fragments mapped to the same regions or windows in sequencing libraries. Input-adjusted short fragments ratio was calculated through dividing the short fragments ratio in investigated regions or genomic windows by the short fragments ratio in whole human reference genome (version hg19) of corresponding Input library. Genome-wide cfDNA fragmentation profiles in Input and IP libraries for participants in discovery cohort were calculated without GC adjustment according to the methods reported in previous study [7].

### Identification of differentially methylated regions (DMRs)

For each sample from participants, we computed cfDNA fragment counts per 10-kb nonoverlapping windows across human reference genome (version hg19), filtered out windows with the mean counts less than 10, and R package DESeq2 (version 1.28.1) with default parameters was used for calling DMRs at padj < 0.05. Hypermethylated and hypomethylated regions were defined as the genomic windows that have log2FoldChange > 1 and log2FoldChange < − 1 in patients with breast cancer compared with healthy individuals, and then illustrated in volcano or heatmap by ggplot2 (version 3.3.2) and pheatmap (version 1.0.12) R packages. Density plot was generated through R package ggplot2 (version 3.3.2) to show fragment size distribution of the cfDNA mapped to hypermethylated and hypomethylated regions. Differentially methylated 10-kb windows were selected as DMRs according to the following criteria. (1) the selected genomic windows should have at least 20 unduplicated cfDNA fragments for all samples including patients with breast cancer and healthy individuals; (2) the selected genomic windows should have input-adjusted short fragments ratio of less than 10 for any samples investigated. For samples from discovery cohort 2, same data processing and analysis were conducted without the above filtering step for identifying DMRs, and DMRs were called at *p* value < 0.05 and |log2FoldChange|> 1. For samples of lung cancer from another study [23], same data processing and analysis were used without deduplication step, and DMRs were called at *p* value < 0.05 and |log2FoldChange|> 1.

### Diagnostic model for breast cancer detection

To distinguish patients with breast cancer from healthy individuals using fragmentation profiles in DMRs, we calculated the median input-adjusted short fragments ratio in each differentially hypomethylated 10-kb windows of healthy individuals in validation cohort (*N* = 8) as a baseline profile. We then evaluated the Pearson correlation of the fragmentation profile in each participants from validation cohort to the baseline profile. Cut-offs threshold was determined as the correlation value that can classify healthy individuals and patients with breast cancer at maximum specificity and sensitivity. Receiver operating characteristic (ROC) curve was used to evaluate the classifiers for predicting breast cancer through the R package pROC (version 1.16.2).

### Supplementary Information

**Additional file 1: Table S1.** Summary of patients and samples analyzed in this study. **Table S2.** Sequenced reads, deduplicated cfDNA fragments in cfMeDIP-seq libraries for patients with breast cancer and healthy individuals. **Table S3.** Altered short cfDNA fragment ratio in patients with breast cancer. **Table S4.** DMRs obtained from cfMeDIP-seq of patients with breast cancer and healthy individuals in discovery cohort 1. **Table S5.** DMRs obtained from cfMeDIP-seq of patients with breast cancer and healthy individuals in validation cohort. **Table S6.** Oligos used in this study.

**Additional file 2: Fig. S1. cfMeDIP-seq library in healthy individuals and patients with breast cancer.** (**A** and **B**) Representative bioanalyzer profile of size distribution in Input library (A) and IP library (B). (**C**) Specificity of the immunoprecipitation reaction and fold-enrichment ratio in sequencing libraries, which were calculated according to the instructions provided by manufacturer. Dots indicated three representatives with horizontal lines representing the mean. (**D**) Yield of cfDNA extracted per ml of plasma from healthy individuals and patients with breast cancer. Horizontal bars represented the mean, dots represented individual samples. (**E**) Amount of cfDNA used for cfMeDIP-seq library construction. **Fig. S2. cfDNA fragmentation in Input library and IP library in discovery cohort 1.** (**A** and **B**) Distribution of cfDNA fragment size were shown for patients with breast cancer (n = 3, purple) and healthy individuals (n = 3, black) in Input library (A) and IP library (B). The vertical dashed line indicated cfDNA fragment size at 100 bp and 150 bp. (**C** and **D**) Short fragments ratio (defined as the ratio of short cfDNA fragments (100 bp—150 bp) to the long cfDNA fragments (151—220 bp)) of Input library (C) and IP library (D) were shown for patients with breast cancer and healthy individuals respectively. (**E**) Percentage change of short fragments ratio in IP libraries compared with corresponding input libraries in patients with breast cancer and healthy individuals. Healthy, healthy individuals; Breast, patients with breast cancer; ** represents P value < 0.01. **Figure S3.** Short cfDNA fragment ratio among DMRs in discovery cohort 2. (A) Volcano plot of DMRs from patients with breast cancer (n = 12) versus healthy individuals (n = 12). Significantly hypermethylated genomic windows were highlighted in red dots with p value < 0.05, log2foldchange > 1, significantly hypomethylated genomic windows were highlighted in blue dots with p value < 0.05 and log2foldchange < -1. (B) Short fragments ratio of cfDNA in hypermethylated and hypomethylated regions in patients with breast cancer and healthy individuals. (C) Percentage change of short fragments ratio in hypomethylated regions compared with it in hypermethylated regions for patients with breast cancer and healthy individuals. Hyper, hypermethylated genomic regions; Hypo, hypomethylated genomic regions; Healthy, healthy individuals; Breast, patients with breast cancer. **Fig. S4. Short cfDNA fragments in lung cancer-related DMRs. (A)** Short fragments ratio of cfDNA in hypermethylated and hypomethylated regions in patients with lung cancer and healthy individuals. H1, H2, and H3 indicated the three healthy individuals in the investigated study; P1, P2, P3, P4, and P5 indicated the five patients with lung cancer in the investigated study. (**B)** Percentage change of short fragments ratio in hypomethylated regions compared with it in hypermethylated regions for patients with breast cancer and healthy individuals. Healthy, healthy individuals; Lung, patients with lung cancer; ** represents P value < 0.01. **Fig. S5. Representation of altered DMRs-dependent cfDNA fragmentation profiles in discovery cohort 1.** (**A**) Input-adjusted short fragments ratio were shown in hypermethylated and hypomethylated regions with 10-kb windows for each patient with breast cancer (upper left text, purple, n = 1) and healthy individuals (black, n = 3). (**B**) The DMRs-dependent cfDNA fragmentation profile mentioned above was shown across human genome. The input-adjusted short fragments ratio in each 10-kb window was calculated by dividing the short fragments ratio in each 10-kb window by the short fragments ratio in corresponding input libraries. Differentially methylated 10-kb windows were selected for representation according to the following criteria: (1) hypermethylated 10-kb windows have padj < 0.05 and log2foldchange > 1; (2) hypomethylated 10-kb windows have padj < 0.05 and log2foldchange < -1; (3) the selected windows should have at least 20 deduplicated cfDNA fragments for all samples including patients with breast cancer and healthy individuals; (4) the selected windows should have input-adjusted short fragments ratio of less than 10 for any samples. Hyper, hypermethylated genomic regions; Hypo, hypomethylated genomic regions. **Fig. S6. cfDNA fragmentation profiles in DMRs in patients with breast cancer.** Input-adjusted short fragments ratio were shown in hypermethylated and hypomethylated regions with 10-kb windows respectively for patients with breast cancer (purple, n = 3) and healthy individuals (black, n = 3). Input-adjusted short fragments ratio in each 10-kb window was calculated by dividing the short fragments ratio in each 10-kb window by the short fragments ratio in corresponding input libraries. Different threshold (upper left text) for defining DMRs were analyzed and shown in separate figures. In addition, windows were selected for representation according to the following criteria: (1) the selected windows should have at least 20 deduplicated cfDNA fragments for all samples including patients with breast cancer and healthy individuals; (2) the selected windows should have input-adjusted short fragments ratio of less than 10 for any samples. Hyper, hypermethylated genomic regions; Hypo, hypomethylated genomic regions. **Fig. S7. cfDNA fragmentation in Input library and IP library in validation cohort.** (**A** and **B**) Distribution of cfDNA fragment size were shown for patients with breast cancer (n = 11, purple) and healthy individuals (n = 8, black) in Input library (A) and IP library (B). The vertical dashed line indicated cfDNA fragment size at 100 bp and 150 bp. Healthy, healthy individuals; Breast, patients with breast cancer. **Fig. S8. Altered fragmentation profiles of methylated cfDNA in validation cohort.** Distribution of cfDNA fragment size in Input library (blue line) and IP library (red line) were shown for healthy individuals (H4, H5, H6, H7, H8, H9, H10, and H11) and patients with breast cancer (P4, P5, P6, P7, P8, P9, P10, P11, P12, P13, and P14). The vertical dashed line indicated cfDNA fragment size at 100 bp and 150 bp. **Fig. S9. Short cfDNA fragment ratio among DMRs in validation cohort. (A)** Volcano plot of DMRs from patients with breast cancer (n = 11) versus healthy individuals (n = 8). Significantly hypermethylated genomic windows were highlighted in red dots with padj < 0.05, log2foldchange > 1, significantly hypomethylated genomic windows were highlighted in blue dots with padj < 0.05 and log2foldchange < -1. (**B)** Heatmap of the 731 DMRs identified in plasma cfDNA from patients with breast cancer and healthy individuals. (**C)** Short fragments ratio of cfDNA in hypermethylated and hypomethylated regions in patients with breast cancer and healthy individuals. (**D)** Percentage change of input-adjusted short fragments ratio in hypomethylated regions when compared with it in hypermethylated regions for patients with breast cancer and healthy individuals. The short fragments ratio in hypermethylated and hypomethylated regions were first adjusted by short fragments ratio in corresponding input libraries, and then the difference were calculated as percentage change in hypomethylated regions compared with hypermethylated regions. Hyper, hypermethylated genomic regions; Hypo, hypomethylated genomic regions; Healthy, healthy individuals; Breast, patients with breast cancer; ** represents P value < 0.01. **Fig. S10. Altered cfDNA fragmentation profiles among hypomethylated regions in validation cohort.** Distribution of cfDNA fragment size were shown for healthy individuals (H4, H5, H6, H7, H8, H9, H10, and H11) and patients with breast cancer (P4, P5, P6, P7, P8, P9, P10, P11, P12, P13, and P14) in hypermethylated regions (red) and hypomethylated regions (green). The vertical dashed line indicated cfDNA fragment size at 100 bp and 150 bp. **Fig. S11. Altered cfDNA fragmentation profiles in hypomethylated regions in each patient with breast cancer in validation cohort.** Input-adjusted short fragments ratio were shown in hypermethylated and hypomethylated regions with 10-kb windows for each patient with breast cancer (upper left text, purple, n = 1) and healthy individuals (black, n = 8). The input-adjusted short fragments ratio in each 10-kb window was calculated by dividing short fragments ratio in each 10-kb window by short fragments ratio in corresponding input libraries. Differentially methylated 10-kb windows were selected for representation according to the following criteria: (1) hypermethylated 10-kb windows have padj < 0.05 and log2foldchange > 1; (2) hypomethylated 10-kb windows have padj < 0.05 and log2foldchange < -1; (3) the selected windows should have at least 20 deduplicated cfDNA fragments for all samples including patients with breast cancer and healthy individuals; (4) the selected windows should have input-adjusted short fragments ratio of less than 10 for any samples. Hyper,hypermethylated genomic regions; Hypo, hypomethylated genomic regions. **Fig. S12. Altered cfDNA fragmentation profiles in hypomethylated regions in each patient with breast cancer across human genome in validation cohort.** Input-adjusted short fragments ratio were shown in hypermethylated and hypomethylated regions across human genome with 10-kb windows for each patient with breast cancer (upper left text, purple, n = 1) and healthy individuals (black, n = 8). The input-adjusted short fragments ratio in each 10-kb window was calculated by dividing short fragments ratio in each 10-kb window by short fragments ratio in corresponding input libraries. Differentially methylated 10-kb windows were selected for representation according to the following criteria: (1) hypermethylated 10-kb windows have padj < 0.05 and log2foldchange > 1; (2) hypomethylated 10-kb windows have padj < 0.05 and log2foldchange < -1; (3) the selected windows should have at least 20 deduplicated cfDNA fragments for all samples including patients with breast cancer and healthy individuals; (4) the selected windows should have input-adjusted short fragments ratio of less than 10 for any samples. Hyper, hypermethylated genomic regions; Hypo, hypomethylated genomic regions.

## Data Availability

Raw sequencing data (fastq) have been deposited in the Genome Sequence Archive in National Genomics Data Center, China National Center for Bioinformation (PRJCA019929, https://ngdc.cncb.ac.cn/bioproject/).
