# Supplementary material for: Altered cfDNA fragmentation profile in hypomethylated regions as diagnostic markers in breast cancer
Source: Epigenetics Chromatin. 2023 Sep 23;16:33. doi: 10.1186/s13072-023-00508-4 (PMC10517480; doi:10.1186/s13072-023-00508-4)
Supplement: Supplementary file 2 — Additional file 2: Fig. S1. cfMeDIP-seq library in healthy individuals and patients with breast cancer. (A and B) Representative bioanalyzer profile of size distribution in Input library (A) and IP library (B). (C) Specificity of the immunoprecipitation reaction and fold-enrichment ratio in sequencing libraries, which were calculated according to the instructions provided by manufacturer. Dots indicated three representatives with horizontal lines representing the mean. (D) Yield of cfDNA extracted per ml of plasma from healthy individuals and patients with breast cancer. Horizontal bars represented the mean, dots represented individual samples. (E) Amount of cfDNA used for cfMeDIP-seq library construction. Fig. S2. cfDNA fragmentation in Input library and IP library in discovery cohort 1. (A and B) Distribution of cfDNA fragment size were shown for patients with breast cancer (n = 3, purple) and healthy individuals (n = 3, black) in Input library (A) and IP library (B). The vertical dashed line indicated cfDNA fragment size at 100 bp and 150 bp. (C and D) Short fragments ratio (defined as the ratio of short cfDNA fragments (100 bp—150 bp) to the long cfDNA fragments (151—220 bp)) of Input library (C) and IP library (D) were shown for patients with breast cancer and healthy individuals respectively. (E) Percentage change of short fragments ratio in IP libraries compared with corresponding input libraries in patients with breast cancer and healthy individuals. Healthy, healthy individuals; Breast, patients with breast cancer; ** represents P value < 0.01. Figure S3. Short cfDNA fragment ratio among DMRs in discovery cohort 2. (A) Volcano plot of DMRs from patients with breast cancer (n = 12) versus healthy individuals (n = 12). Significantly hypermethylated genomic windows were highlighted in red dots with p value < 0.05, log2foldchange > 1, significantly hypomethylated genomic windows were highlighted in blue dots with p value < 0.05 and log2foldchange < -1. (B) Shor [file 13072_2023_508_MOESM2_ESM.docx]

Supplementary Information for

**Altered cfDNA fragmentation profile in hypomethylated regions as diagnostic markers in breast cancer**

Jun Wang, Yanqin Niu, Ming Yang, Lirong Shu, Hongxian Wang, Xiaoqian Wu, Yaqin He, Peng Chen, Guocheng Zhong, Zhixiong Tang, Shasha Zhang, Qianwen Guo, Yun Wang, Li Yu, Deming Gou*

*Corresponding author. Email: dmgou@szu.edu.cn (D.G.)

**This PDF file includes:**

Figs. S1 to S12

**Additional Figures**


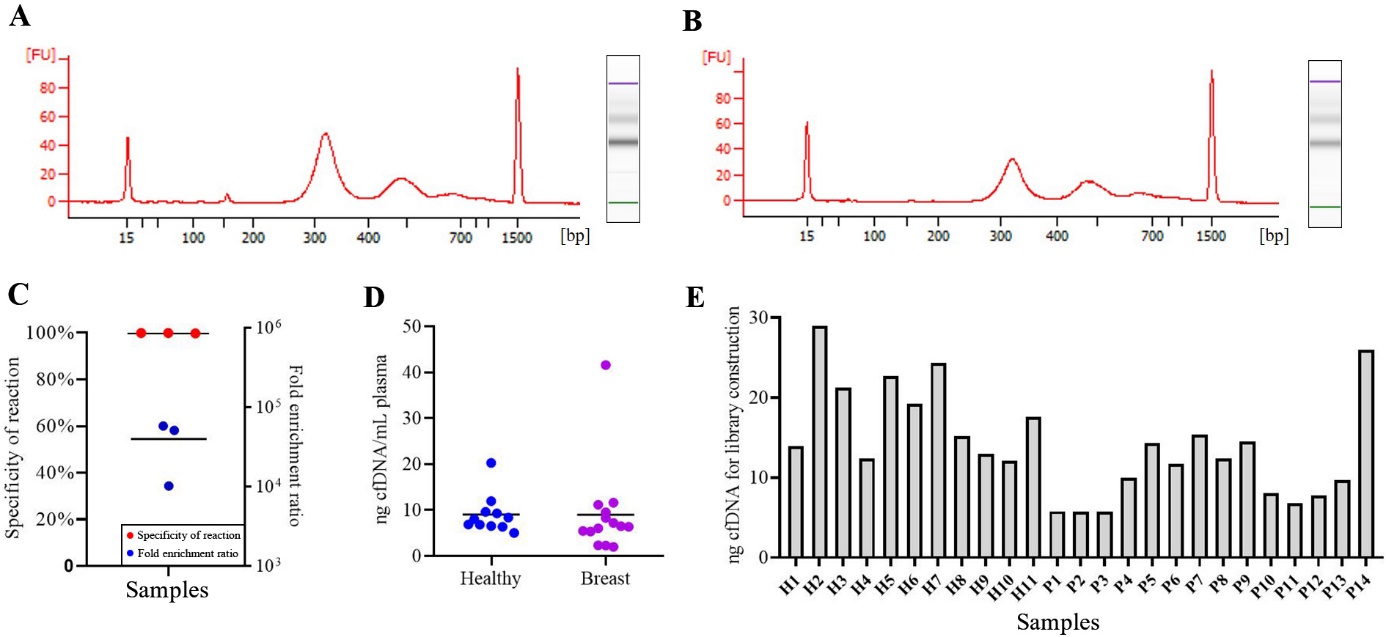


**Fig. S1. cfMeDIP-seq library in healthy individuals and patients with breast cancer.** (**A** and **B**) Representative bioanalyzer profile of size distribution in Input library (A) and IP library (B). (**C**) Specificity of the immunoprecipitation reaction and fold-enrichment ratio in sequencing libraries, which were calculated according to the instructions provided by manufacturer. Dots indicated three representatives with horizontal lines representing the mean. (**D**) Yield of cfDNA extracted per ml of plasma from healthy individuals and patients with breast cancer. Horizontal bars represented the mean, dots represented individual samples. (**E**) Amount of cfDNA used for cfMeDIP-seq library construction.


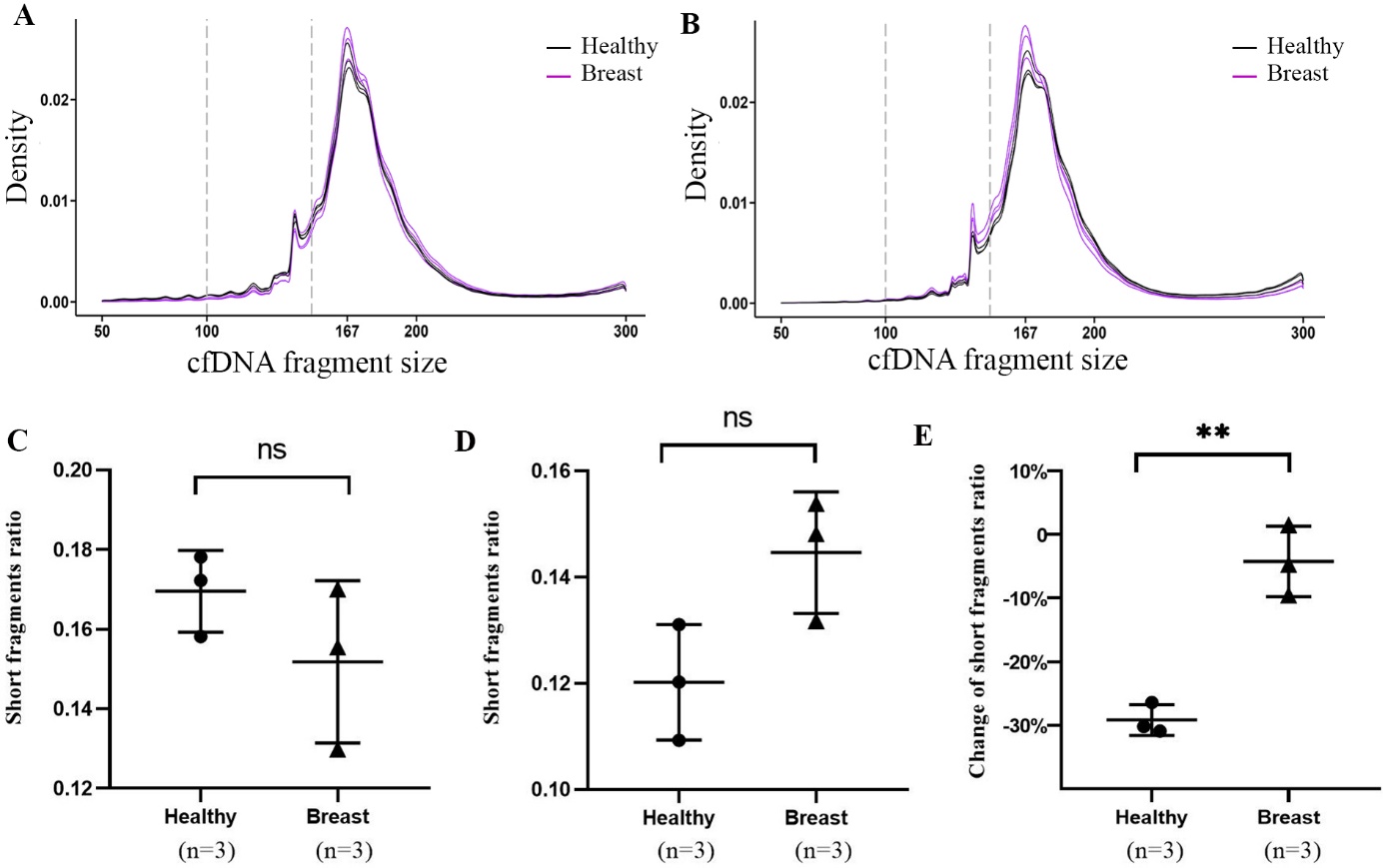


**Fig. S2. cfDNA fragmentation in Input library and IP library in discovery cohort 1.** (**A** and **B**) Distribution of cfDNA fragment size were shown for patients with breast cancer (n = 3, purple) and healthy individuals (n = 3, black) in Input library (A) and IP library (B). The vertical dashed line indicated cfDNA fragment size at 100bp and 150bp. (**C** and **D**) Short fragments ratio (defined as the ratio of short cfDNA fragments (100bp - 150bp) to the long cfDNA fragments (151 - 220bp)) of Input library (C) and IP library (D) were shown for patients with breast cancer and healthy individuals respectively. (**E**) Percentage change of short fragments ratio in IP libraries compared with corresponding input libraries in patients with breast cancer and healthy individuals. Healthy, healthy individuals; Breast, patients with breast cancer; ** represents P value < 0.01.

**
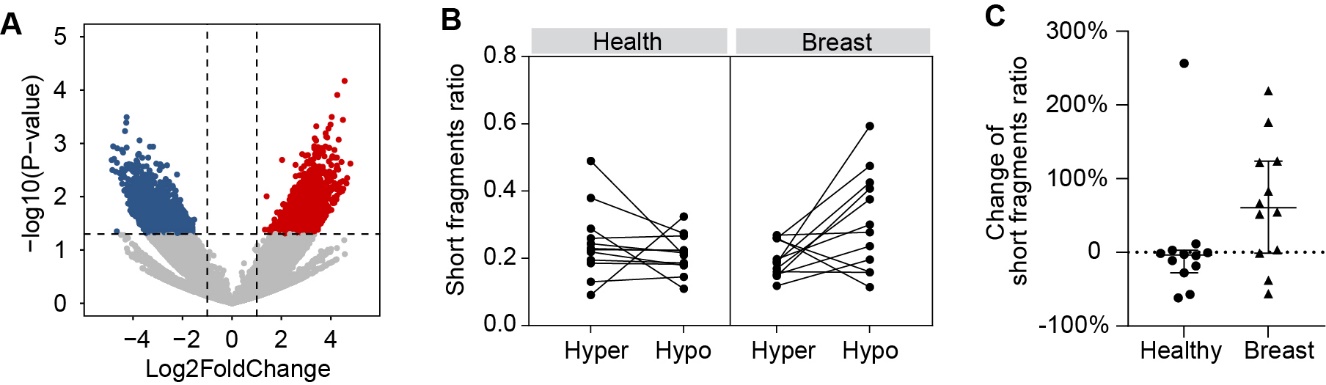
**

**Figure S3.** Short cfDNA fragment ratio among DMRs in discovery cohort 2. (A) Volcano plot of DMRs from patients with breast cancer (n = 12) versus healthy individuals (n = 12). Significantly hypermethylated genomic windows were highlighted in red dots with pvalue < 0.05, log2foldchange > 1, significantly hypomethylated genomic windows were highlighted in blue dots with pvalue < 0.05 and log2foldchange < -1. (B) Short fragments ratio of cfDNA in hypermethylated and hypomethyled regions in patients with breast cancer and healthy individuals. (C) Percentage change of short fragments ratio in hypomethylated regions compared with it in hypermethylated regions for patients with breast cancer and healthy individuals. Hyper, hypermethylated genomic regions; Hypo, hypomethylated genomic regions; Healthy, healthy individuals; Breast, patients with breast cancer.


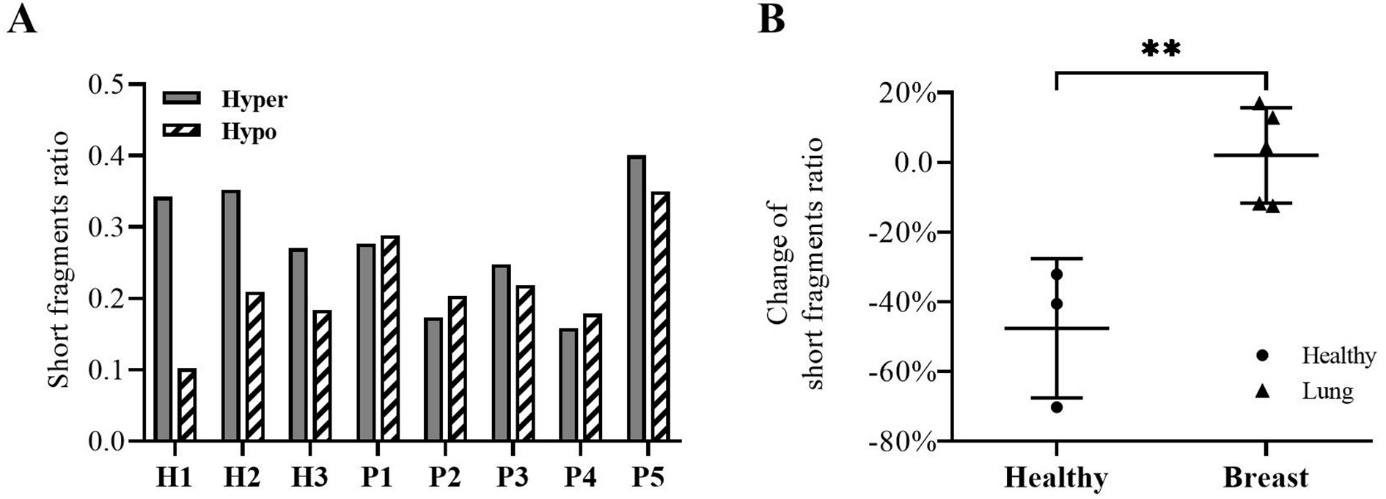


**Fig. S4. Short cfDNA fragments in lung cancer-related DMRs. (A)** Short fragments ratio of cfDNA in hypermethylated and hypomethyled regions in patients with lung cancer and healthy individuals. H1, H2 and H3 indicated the three healthy individuals in the investigated study; P1, P2, P3, P4 and P5 indicated the five patients with lung cancer in the investigated study. (**B)** Percentage change of short fragments ratio in hypomethylated regions compared with it in hypermethylated regions for patients with breast cancer and healthy individuals. Healthy, healthy individuals; Lung, patients with lung cancer; ** represents P value < 0.01.


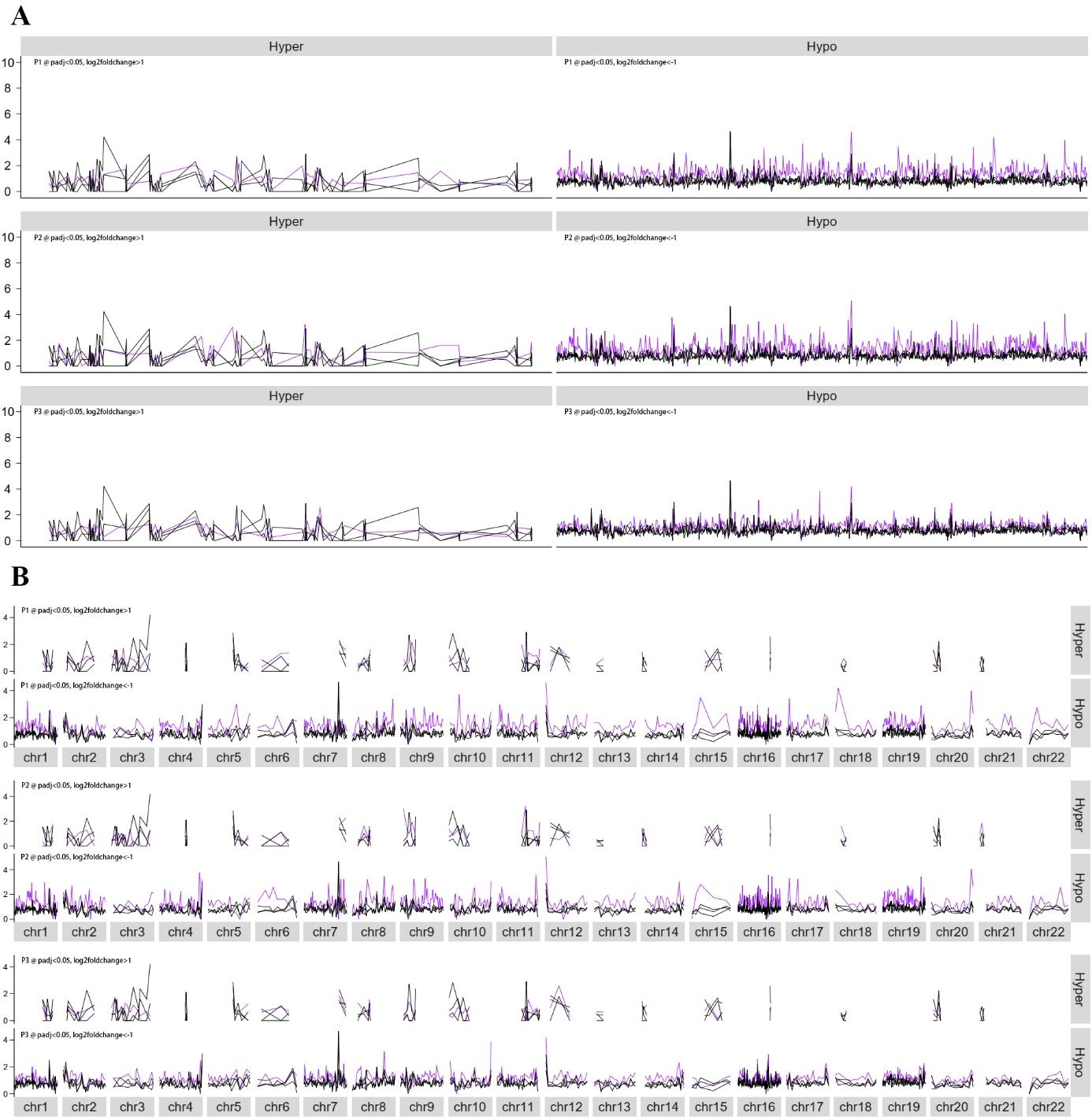


**Fig. S5. Representation of altered DMRs-dependent cfDNA fragmentation profiles in discovery cohort 1.** (**A**) Input-adjusted short fragments ratio were shown in hypermethylated and hypomethylated regions with 10-kb windows for each patient with breast cancer (upper left text, purple, n = 1) and healthy individuals (black, n = 3). (**B**) The DMRs-dependent cfDNA fragmentation profile mentioned above was shown across human genome. The input-adjusted short fragments ratio in each 10-kb window was calculated by dividing the short fragments ratio in each 10-kb window by the short fragments ratio in corresponding input libraries. Differentially methylated 10-kb windows were selected for representation according to the following criteria: (1) hypermethylated 10-kb windows have padj < 0.05 and log2foldchange > 1; (2) hypomethylated 10-kb windows have padj < 0.05 and log2foldchange <-1; (3) the selected windows should have at least 20 deduplicated cfDNA fragments for all samples including patients with breast cancer and healthy individuals; (4) the selected windows should have input-adjusted short fragments ratio of less than 10 for any samples. Hyper, hypermethylated genomic regions; Hypo, hypomethylated genomic regions.


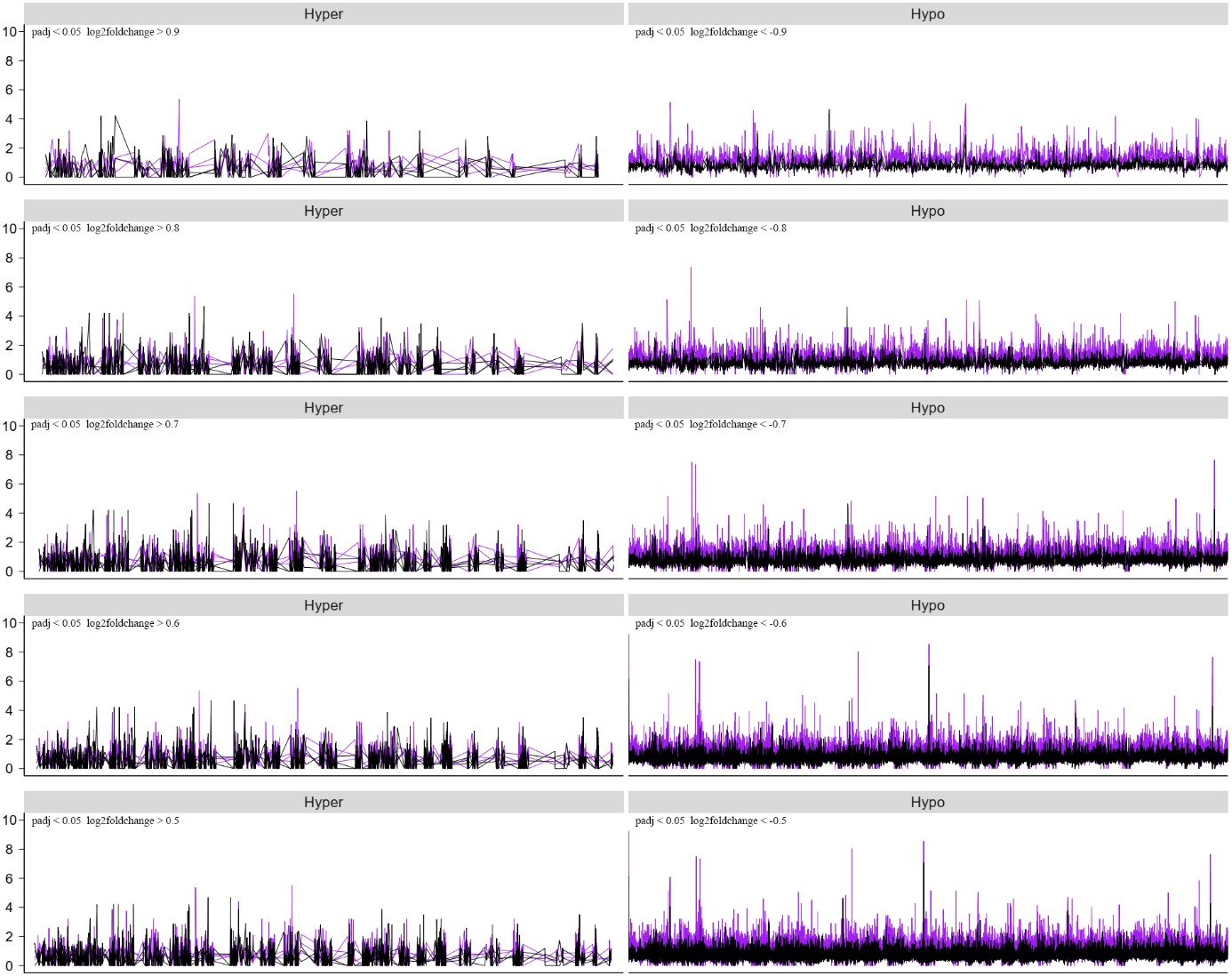


**Fig. S6. cfDNA fragmentation profiles in DMRs in patients with breast cancer.** Input-adjusted short fragments ratio were shown in hypermethylated and hypomethylated regions with 10-kb windows respectively for patients with breast cancer (purple, n = 3) and healthy individuals (black, n = 3). Input-adjusted short fragments ratio in each 10-kb window was calculated by dividing the short fragments ratio in each 10-kb window by the short fragments ratio in corresponding input libraries. Different threshold (upper left text) for defining DMRs were analysed and shown in separate figures. In addition, windows were selected for representation according to the following criteria: (1) the selected windows should have at least 20 deduplicated cfDNA fragments for all samples including patients with breast cancer and healthy individuals; (2) the selected windows should have input-adjusted short fragments ratio of less than 10 for any samples. Hyper, hypermethylated genomic regions; Hypo, hypomethylated genomic regions.


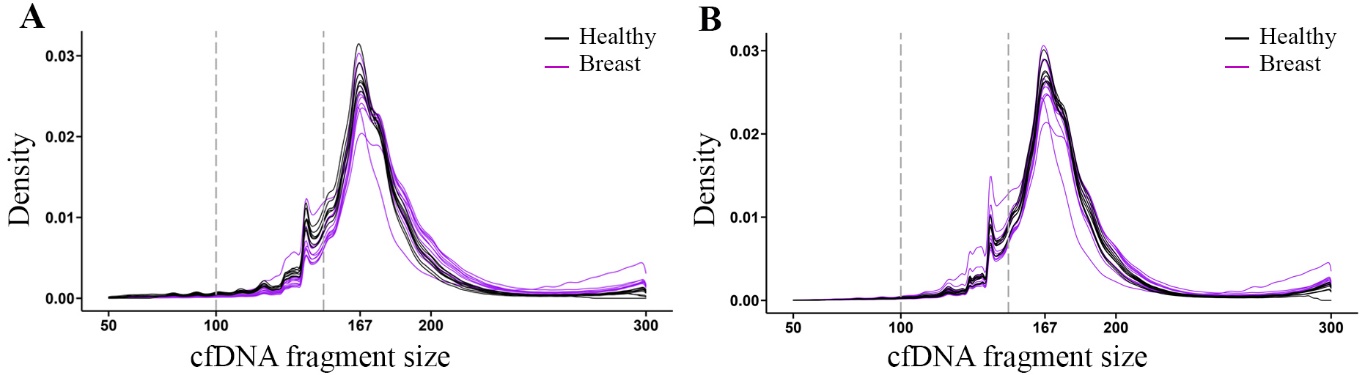


**Fig. S7. cfDNA fragmentation in Input library and IP library in validation cohort.** (**A** and **B**) Distribution of cfDNA fragment size were shown for patients with breast cancer (n = 11, purple) and healthy individuals (n = 8, black) in Input library (A) and IP library (B). The vertical dashed line indicated cfDNA fragment size at 100bp and 150bp. Healthy, healthy individuals; Breast, patients with breast cancer.

**
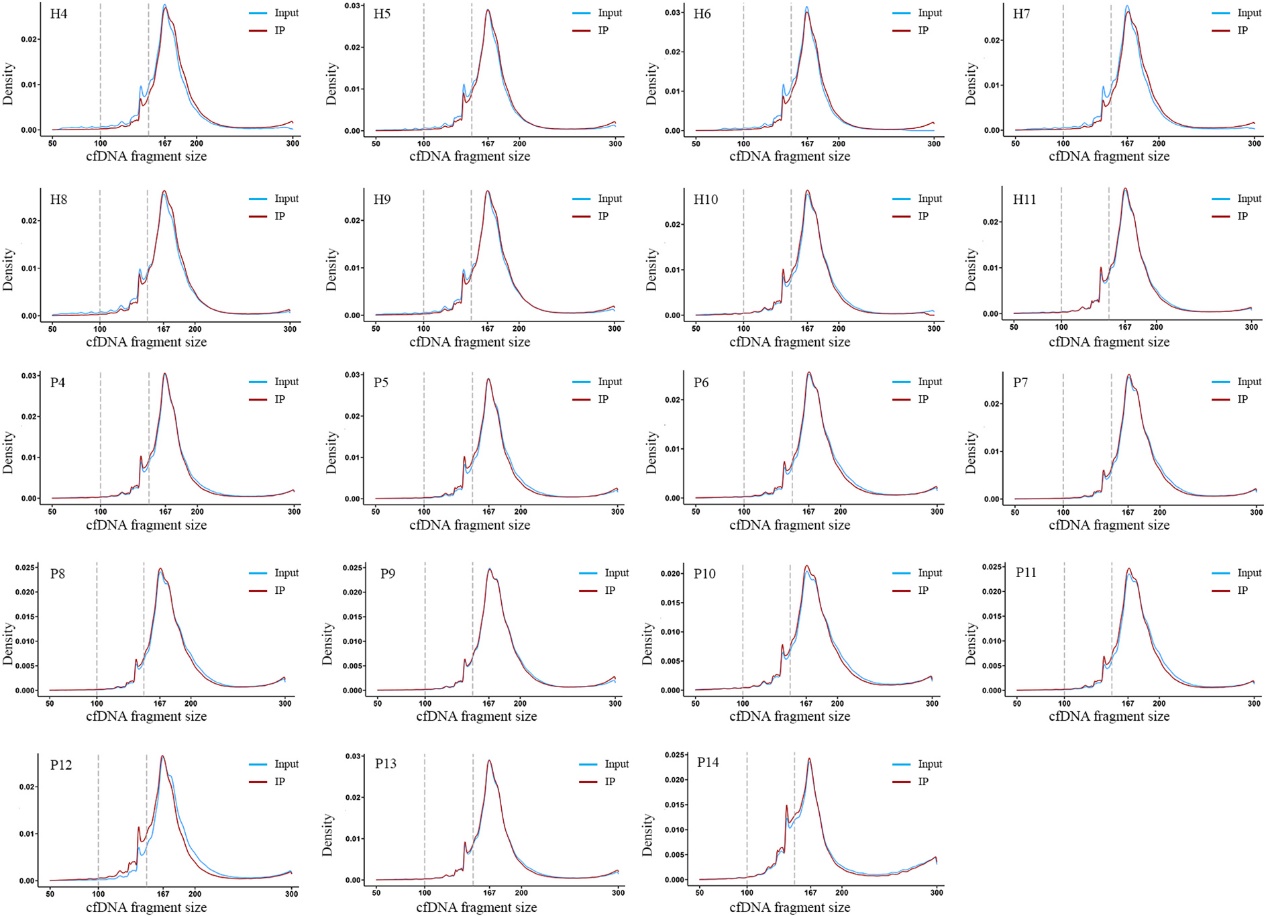
**

**Fig. S8. Altered fragmentation profiles of methylated cfDNA in validation cohort.** Distribution of cfDNA fragment size in Input library (blue line) and IP library (red line) were shown for healthy individuals (H4, H5, H6, H7, H8, H9, H10 and H11) and patients with breast cancer (P4, P5, P6, P7, P8, P9, P10, P11, P12, P13 and P14). The vertical dashed line indicated cfDNA fragment size at 100bp and 150bp.


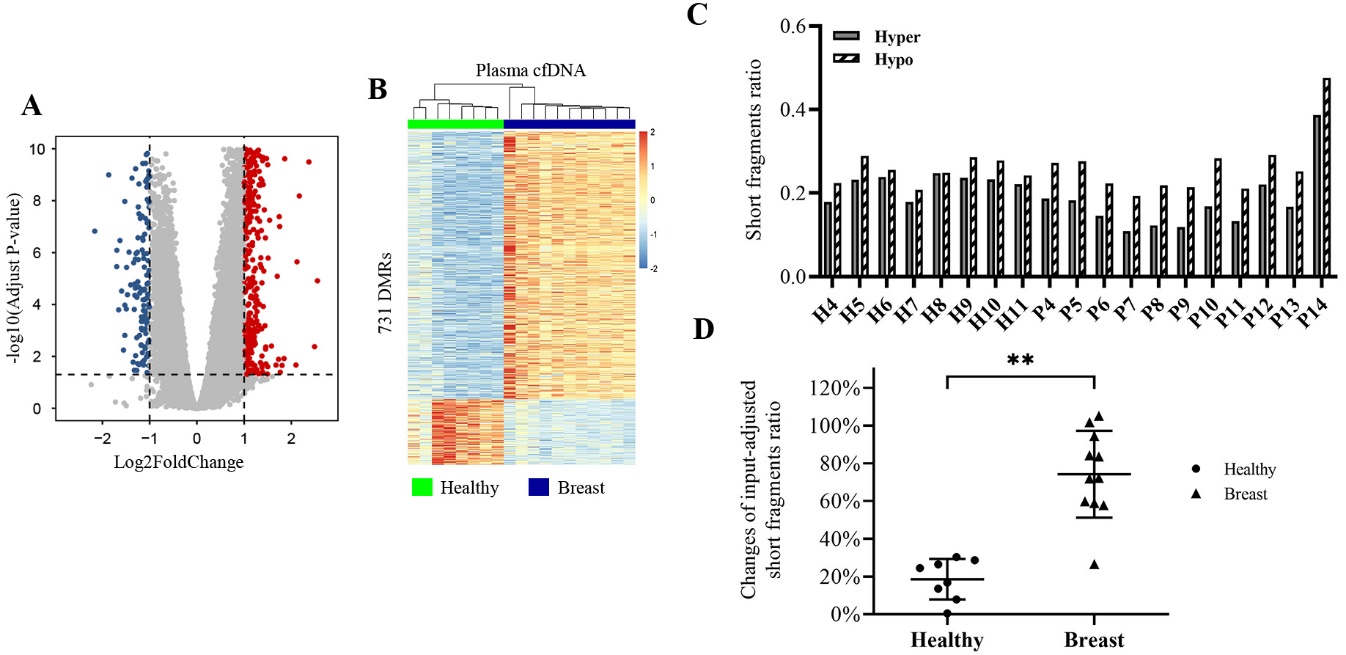


**Fig. S9. Short cfDNA fragment ratio among DMRs in validation cohort. (A)** Volcano plot of DMRs from patients with breast cancer (n = 11) versus healthy individuals (n = 8). Significantly hypermethylated genomic windows were highlighted in red dots with padj < 0.05, log2foldchange > 1, significantly hypomethylated genomic windows were highlighted in blue dots with padj < 0.05 and log2foldchange < -1. (**B)** Heatmap of the 731 DMRs identified in plasma cfDNA from patients with breast cancer and healthy individuals. (**C)** Short fragments ratio of cfDNA in hypermethylated and hypomethyled regions in patients with breast cancer and healthy individuals. (**D)** Percentage change of input-adjusted short fragments ratio in hypomethylated regions compared with it in hypermethylated regions for patients with breast cancer and healthy individuals. The short fragments ratio in hypermethylated and hypomethylated regions were first adjusted by short fragments ratio in corresponding input libraries, and then the difference were calculated as percentage change in hypomethylaed regions compared with hypermethylated regions. Hyper, hypermethylated genomic regions; Hypo, hypomethylated genomic regions; Healthy, healthy individuals; Breast, patients with breast cancer; ** represents P value < 0.01.

**
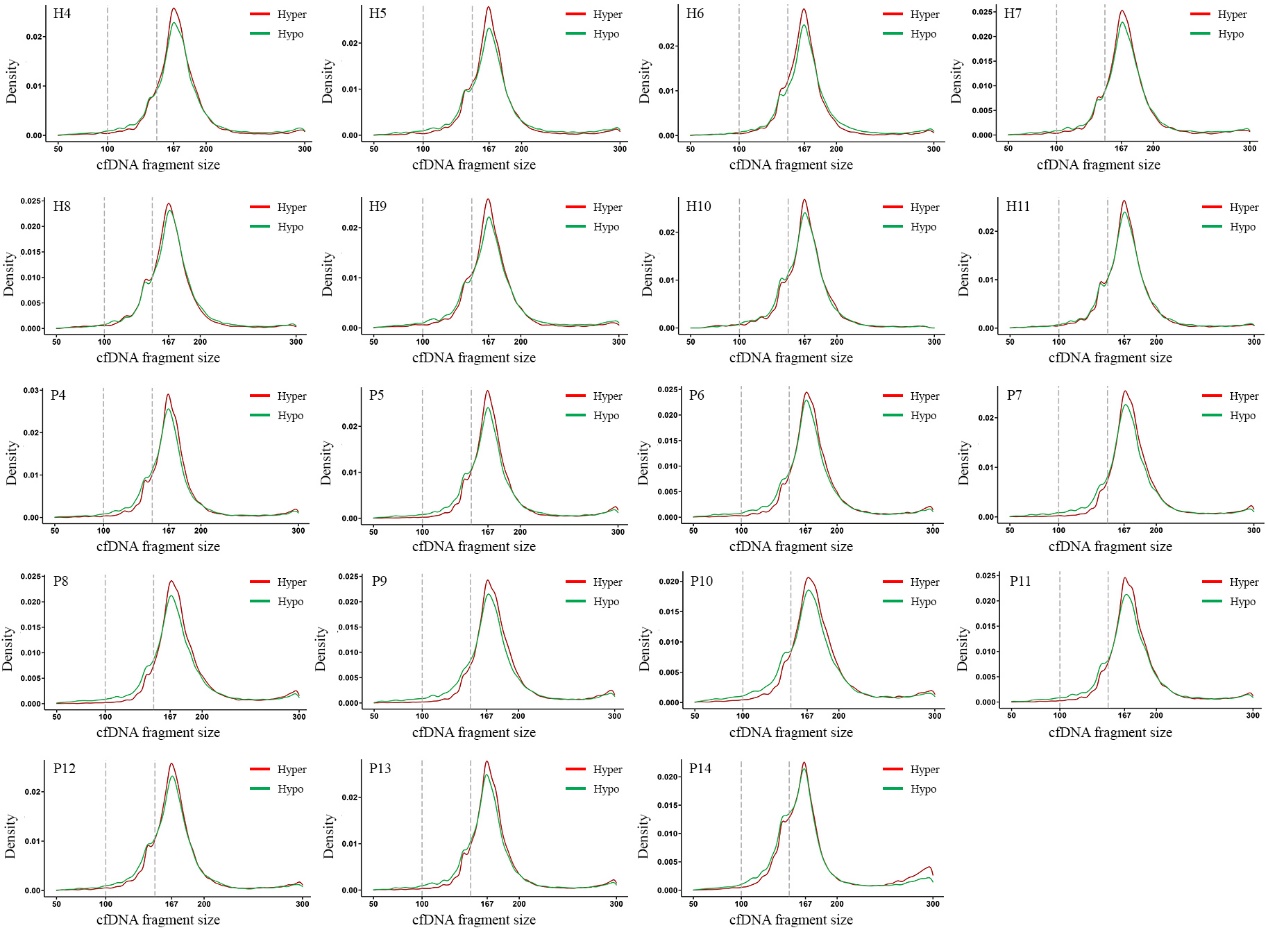
**

**Fig. S10. Altered cfDNA fragmentation profiles among hypomethylated regions in validation cohort.** Distribution of cfDNA fragment size were shown for healthy individuals (H4, H5, H6, H7, H8, H9, H10 and H11) and patients with breast cancer (P4, P5, P6, P7, P8, P9, P10, P11, P12, P13 and P14) in hypermethylated regions (red) and hypomethylated regions (green). The vertical dashed line indicated cfDNA fragment size at 100bp and 150bp.

**
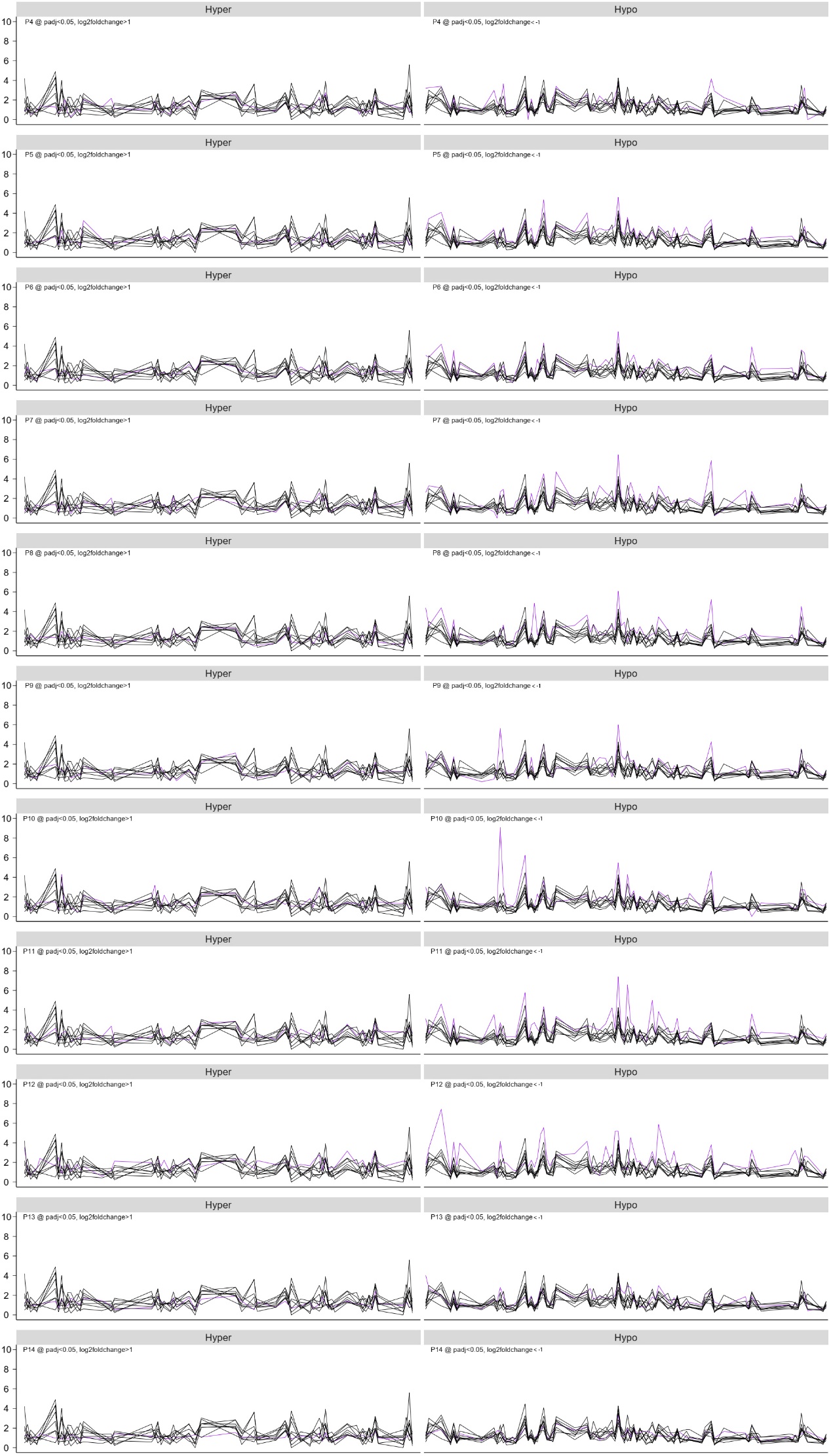
**

**Fig. S11. Altered cfDNA fragmentation profiles in hypomethylated regions in each patient with breast cancer in validation cohort.** Input-adjusted short fragments ratio were shown in hypermethylated and hypomethylated regions with 10-kb windows for each patient with breast cancer (upper left text, purple, n =1) and healthy individuals (black, n = 8). The input-adjusted short fragments ratio in each 10-kb window was calculated by dividing short fragments ratio in each 10-kb window by short fragments ratio in corresponding input libraries. Differentially methylated 10-kb windows were selected for representation according to the following criteria: (1) hypermethylated 10-kb windows have padj < 0.05 and log2foldchange > 1; (2) hypomethylated 10-kb windows have padj < 0.05 and log2foldchange < -1; (3) the selected windows should have at least 20 deduplicated cfDNA fragments for all samples including patients with breast cancer and healthy individuals; (4) the selected windows should have input-adjusted short fragments ratio of less than 10 for any samples. Hyper, hypermethylated genomic regions; Hypo, hypomethylated genomic regions.

**
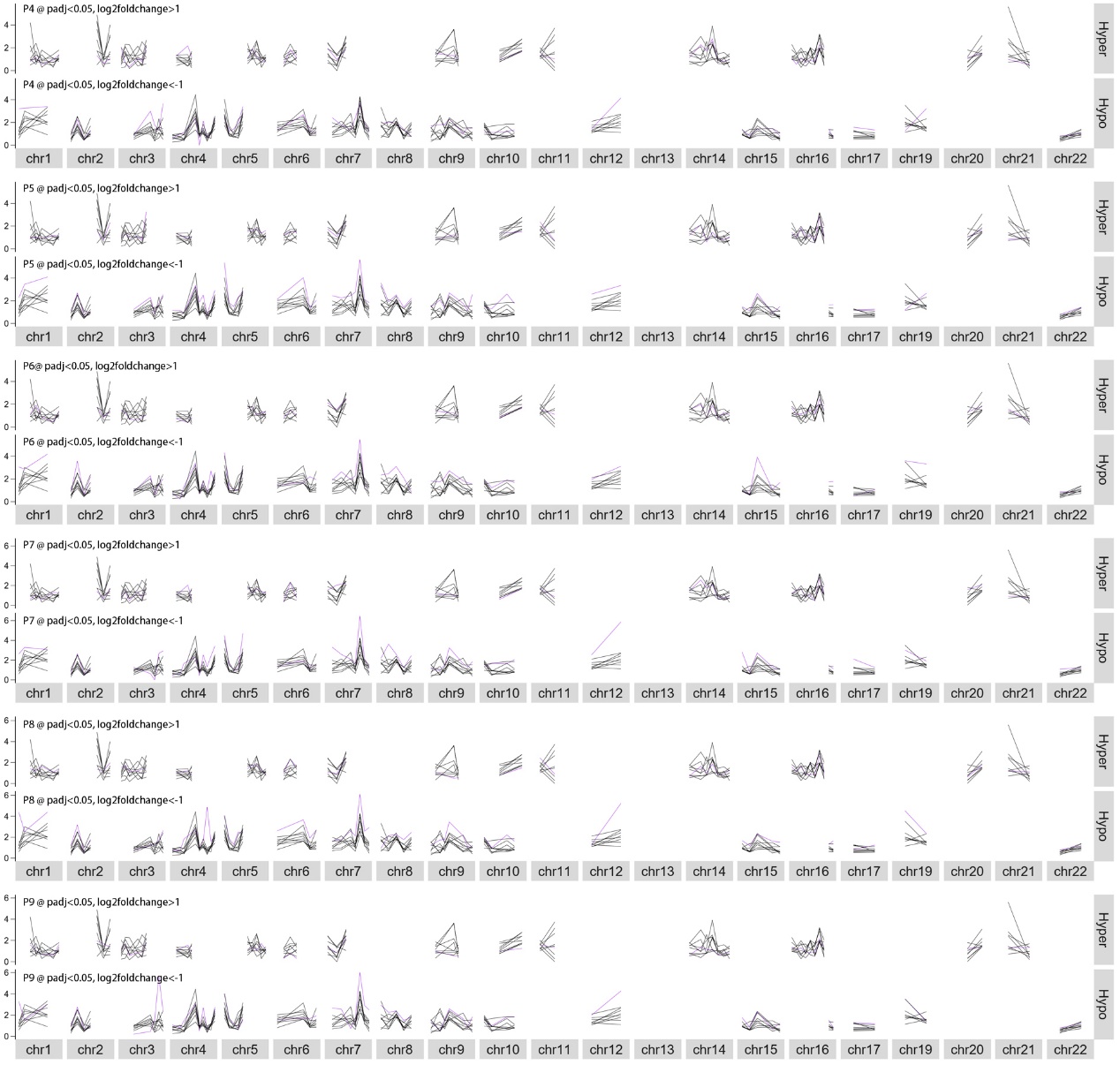
**

**(continue)**

**
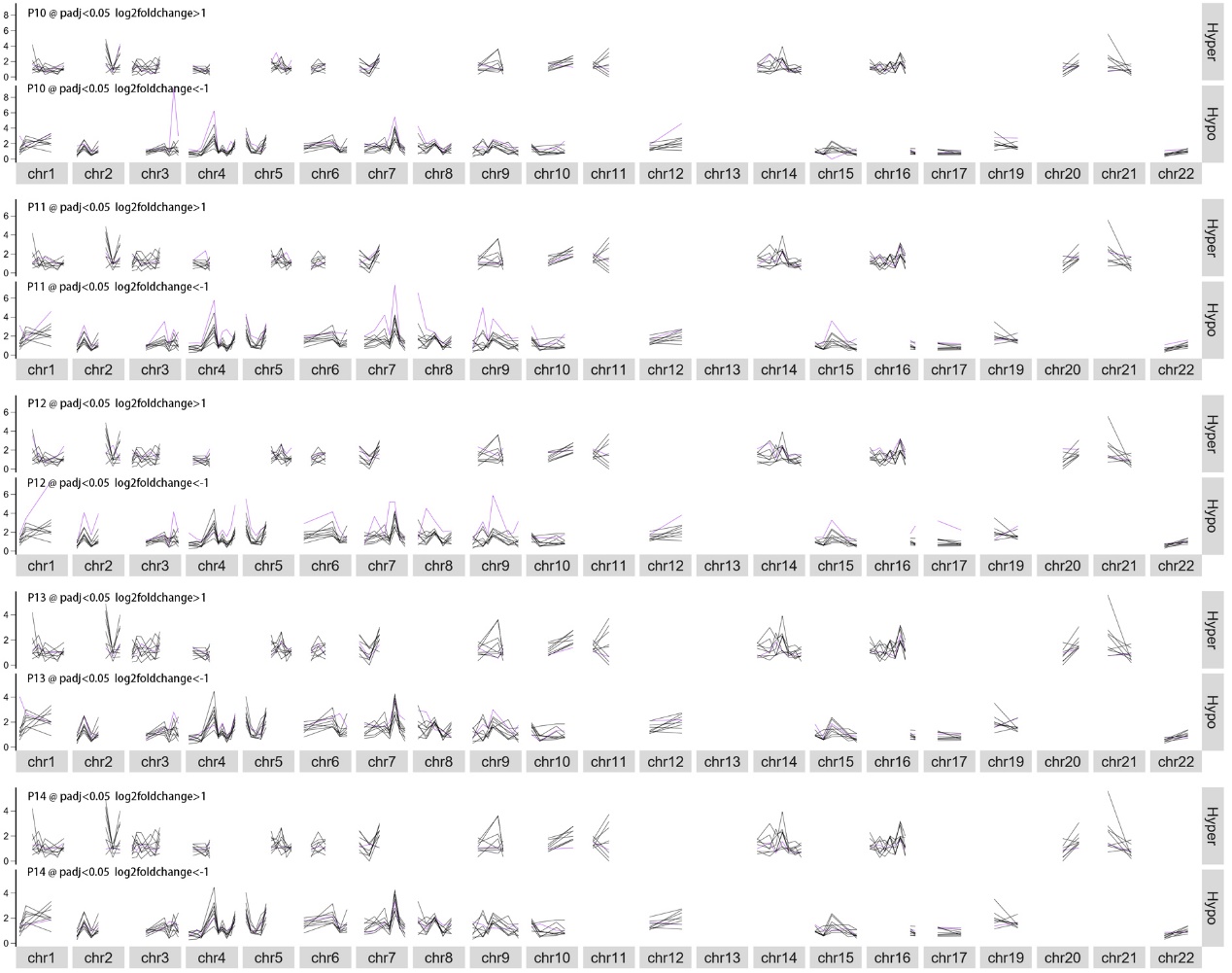
**

**Fig. S12. Altered cfDNA fragmentation profiles in hypomethylated regions in each patient with breast cancer across human genome in validation cohort.** Input-adjusted short fragments ratio were shown in hypermethylated and hypomethylated regions across human genome with 10-kb windows for each patient with breast cancer (upper left text, purple, n =1) and healthy individuals (black, n = 8). The input-adjusted short fragments ratio in each 10-kb window was calculated by dividing short fragments ratio in each 10-kb window by short fragments ratio in corresponding input libraries. Differentially methylated 10-kb windows were selected for representation according to the following criteria: (1) hypermethylated 10-kb windows have padj < 0.05 and log2foldchange > 1; (2) hypomethylated 10-kb windows have padj < 0.05 and log2foldchange < -1; (3) the selected windows should have at least 20 deduplicated cfDNA fragments for all samples including patients with breast cancer and healthy individuals; (4) the selected windows should have input-adjusted short fragments ratio of less than 10 for any samples. Hyper, hypermethylated genomic regions; Hypo, hypomethylated genomic regions.
